# Discovery of Novel Anti-cryptosporidial Activities From Natural Products by *in vitro* High-Throughput Phenotypic Screening

**DOI:** 10.3389/fmicb.2019.01999

**Published:** 2019-08-29

**Authors:** Zi Jin, Jingbo Ma, Guan Zhu, Haili Zhang

**Affiliations:** ^1^Department of Veterinary Pathobiology, College of Veterinary Medicine & Biomedical Sciences, Texas A&M University, College Station, TX, United States; ^2^Department of Parasitology, School of Basic Medical Sciences, Xinxiang Medical University, Xinxiang, China

**Keywords:** apicomplexan, *Cryptosporidium parvum*, drug discovery, natural products, high-throughput screening

## Abstract

*Cryptosporidium parvum* is a globally distributed zoonotic protozoan parasite of both medical and veterinary importance. Nitazoxanide is the only FDA-approved drug to treat cryptosporidiosis in immunocompetent people, but it is not fully effective. There is no drug approved by FDA for use in immunocompromised patients or in animals. In the present study, we conducted phenotypic screening of 800 nature products with defined chemical structures for potential novel activity against the growth of *C. parvum in vitro*. We identified a large number of compounds showing low to sub-micromolar anti-cryptosporidial activity, and fully characterized 16 top hits for anti-parasitic efficacies *in vitro* [EC_50_ values from 0.122 to 3.940 μM, cytotoxicity (TC_50_) values from 6.31 to >100 μm] and their safety margins. Among them, 11 compounds were derived from plants with EC_50_ values from 0.267 to 3.940 μM [i.e., cedrelone, deoxysappanone B 7,4′-dimethyl ether (Deox B 7,4), tanshinone IIA, baicalein, deoxysappanone B 7,3′-dimethyl ether acetate, daunorubicin, dihydrogambogic acid, deacetylgedunin, deacetoxy-7-oxogedunin, dihydrotanshinone I, 2,3,4′-trihydroxy-4-methoxybenzophenone, and 3-deoxo-3beta-hydroxy-mexicanolide 16-enol ether]. Three compounds with sub-micromolar EC_50_ values (i.e., cedrelone, Deox B 7,4, and baicalein) were further investigated for their effectiveness on various parasite developmental stages *in vitro*. Cedrelone and baicalein were more effective than Dexo B 7,4 when treating parasite for shorter periods of time, but all three compounds could kill the parasite irreversibly. These findings provide us a large selection of new structures derived from natural products to be explored for developing anti-cryptosporidial therapeutics.

## Introduction

*Cryptosporidium parvum* (Phylum Apicomplexa) is a zoonotic protozoan parasite causing cryptosporidiosis in humans and animals. In humans, *C. parvum* infection can result in mild to severe watery diarrhea that is usually self-resolved in a couple of weeks in immunocompetent individuals, but could be chronic and deadly in immunocompromised patients (Goodgame, 2003; Kotloff et al., 2013; Miyamoto and Eckmann, 2015). In developing countries, *Cryptosporidium* is one of the top agents causing diarrhea and associated with stunted growth and increased fatality in children (Molbak et al., 1997; Checkley et al., 1998; Mondal et al., 2009; Kotloff et al., 2013; Mmbaga and Houpt, 2017). It is also an important water-borne and food-borne pathogen, frequently causing cryptosporidiosis outbreaks around world. In the United States alone, there was an earlier estimation of 748,000 annual cases of cryptosporidiosis (Scallan et al., 2011; Painter et al., 2015). Historically, the number of reported cases of cryptosporidiosis in the United States caused by contaminated water and food increased from 7,656 in 2009 to 9,313 in 2011; and then decreased to 8,008 in 2012 (Yoder et al., 2012; Painter et al., 2015). A more recent analysis of 7,465 cases in the period of 2009–2017 in the United States showed that ingestion of recreational water (e.g., pools and water playgrounds) was the predominant risk factors responsible for 35.1% outbreaks and 56.7% cases (Gharpure et al., 2019). In farm animals, *Cryptosporidium* is an important pathogen responsible for the neonatal diarrhea syndrome of calves, lambs and other young ruminants, resulting in considerable direct, and indirect economic losses (De Graaf et al., 1999). However, current treatment options for cryptosporidiosis are limited (Bessoff et al., 2014). No drugs are FDA-approved for treating cryptosporidiosis in animals in the United States, while nitazoxanide (NTZ) is the only drug approved by FDA for treating cryptosporidial infection in immunocompetent human patients, but not in people with compromised immunity (Bailey and Erramouspe, 2004; Rossignol et al., 2006; Abubakar et al., 2007; Amadi et al., 2009; Chavez and White, 2018).

Besides NTZ, a few other marketed drugs including paromomycin and azithromycin possess certain levels of anti-cryptosporidial activity, but they are not FDA-approved for treating cryptosporidiosis (Holmberg et al., 1998; Allam and Shehab, 2002; Bessoff et al., 2013; Madbouly Taha et al., 2017). More recently, several promising anti-cryptosporidial lead compounds have been reported with low nanomolar activity *in vitro* and low mg/kg activity in animal models, such as the compound 1294 acting on calcium dependent protein kinase (CDPK) (Castellanos-Gonzalez et al., 2013), P131 on inosine-5′-mono-phosphate dehydrogenase IMPDH (Gorla et al., 2014), KDU731 on phosphatidylinositol-4-OH kinase [PI(4)K] (Manjunatha et al., 2017), triacsin C on acyl-CoA synthetase (ACS) (Guo et al., 2014), and SAHA on histone-deacetylase (HDAC) (Guo et al., 2018). They are still in the lead optimization or pre-clinical stages of development. Therefore, there is an urgent need to discover new anti-cryptosporidial compounds, particularly those with the potential for use in young and immunocompromised patients and animals (Checkley et al., 2015).

Natural products are virtually an unlimited source of highly diversified chemical structures for discovering various medicinal activities. Many antibiotics and drugs are in fact natural products, such as camptothecin, lovastatin, quinine, and silibinin (Tran et al., 1996; Lacombe et al., 2014; De Oliveira et al., 2015; Feng et al., 2018). The Nobel Prize-winning anti-malarial drug, artemisinin (qinghaosu), is a natural product from plant *Artemisia annua L.* (Kong and Tan, 2015). Due to the lack of high-throughput screening (HTS) system in earlier days, there had been only a few reports on evaluating of anti-cryptosporidial activity of selected non-microbial natural products or plant extracts [e.g., (Kim and Healey, 2001; Maruyama et al., 2007; Shahiduzzaman et al., 2009; Teichmann et al., 2012, 2016; Weyl-Feinstein et al., 2014; Ch Stratakos et al., 2017)].

In the present study, we took advantage of our recently developed qRT-PCR assay for HTS of anti-cryptosporidial drugs (Zhang and Zhu, 2015), and screened a total of 800 structurally diverse natural products for their activities against the growth of *C. parvum in vitro*. We discovered more than 16 natural products showing low to sub-micromolar anti-*C. parvum* activity, and analyzed the action of 3 compounds for their activity on various developmental stages of *C. parvum* in more detail. Our findings provide a set of new chemical structures as anti-cryptosporidial hits for further investigations.

## Materials and Methods

### *In vitro* Culture of *C. parvum*

The culture of *C. parvum in vitro* and drug screening were performed as described (Zhang and Zhu, 2015; Guo et al., 2018). Briefly, fresh oocysts of *C. parvum* (BGF-1 strain; subtype IIaA17G2R1) were purchased from Bunch Grass Farm (Deary, ID, United States), purified using a Percoll-based gradient centrifugation method and sterilized with 10% bleach for 7 min on ice, followed by extensive washes with phosphate-buffered saline (PBS). Oocysts less than 3 months old since harvest were used in all experiments. The strain of *C. parvum* was originally described as Iowa-1 strain (subtype IIaA15G2R1), but has been replaced by a new strain with a subtype IIaA17G2R1. For clarity, we have renamed it as BGF-1 strain.

Host cells used HCT-8 cell line derived from a human ileocecal adenocarcinoma (American Type Culture Collection # CCL-225). HCT-8 cells were seeded in 96-well plate at a density of 23,000 cells/well and allowed to grow overnight in 200 μL RPMI-1640 medium with 10% fetal bovine serum (FBS) at 37°C under 5% CO_2_ atmosphere. After cell monolayers reached to 80–90% confluence, plates were incubated with *C. parvum* oocysts (20,000 oocysts/well) for 3 h, and uninvaded parasites were removed by a medium exchange. Compounds at designed concentration and diluent were added at this point. Parasite-infected cells were cultured for additional 41 h (total 44 h infection time) and lysed at this time point for qRT-PCR as described below.

### qRT-PCR Assay

The relative levels of *C. parvum* were determined by detecting the levels of 18S rRNA transcripts (Cp18S), normalized with those of host cell 18S rRNA transcripts (Hs18S) by qRT-PCR. After 44 h post-infection (hpi) time, plates were gently washed 3 times with PBS, followed by the addition of 150 μL ice-cold Bio-Rad iScript qRT-PCR sample preparation regent (lysis buffer) (Bio-Rad Laboratories, Hercules, CA, United States). Plates were sealed with adhesive and heat-sealing films and vortexed in a bucket containing ice for 20 min in a plate vortexer (VX-2500, VWR International, Radnor, PA, United States; speed at 7). The plates were centrifuged (5 min, 2000 × *g*) to ensure all the debris were attached to the bottom of the wells and then incubated at 75°C for 15 min. The supernatants are either stored at −80°C or directly used for qRT-PCR.

For qRT-PCR detection, the lysates were diluted by 100 and 2000 folds for detecting Cp18S and Hs18S transcripts, respectively, using a qScript one-step SYBR green qRT-PCR kit (Quanta Biosciences, Gaithersbury, MD, United States). Hs18S levels were used as controls and for normalization. The qRT-PCR reactions were carried out in 384-well plates in a CFX384 Touch Real-Time PCR Detection System (Bio-Rad Laboratories). Each well contained 10 μL reaction solution mixed with 3 μL diluted cell lysate, 5 μL one-step SYBR green master mix, 0.2 μL RT master mix, and primers for Cp18S (i.e., Cp18S-1011F, 5′-TTG TTC CTT ACT CCT TCA GCA C-3′ and Cp18S-1185R, 5′- TCC TTC CTA TGT CTG GAC CTG-3′; 200 nM) or Hs18S transcripts (i.e., Hs18S-1F, 5′-GGC GCC CCC TCG ATG CTC TTA-3′ and Hs18S-1R, 5′-CCC CCG GCC GTC CCT CTT A-3′; 700 nM). The cycle threshold (C_T_) were recorded for computing relative parasite loads based on ΔΔC_T_ values.

During the course of this study, standard curves derived from specimens infected with various numbers of *C. parvum* were produced for new batches of parasites and reagents for assessing the PCR amplification efficiency, i.e., calculation of the parameter A by linear regression between C_T_ values and the logarithm of inoculated oocyst numbers (A = 1/Slope). A percent inhibition of parasite growth could be calculated using following equation as described (Cai et al., 2005; Zhang and Zhu, 2015):

(1) $$Inhibition\left(\%\right)=\left(1-10^{A\,\Delta \,\Delta \,C_{T}}\right)\cdot 100$$

In comparison with the following simplified empirical equation that assumes perfect PCR amplification efficiency:

(2) $$Inhibition\left(\%\right)=\left(1-2^{-\Delta \,\Delta \,C_{T}}\right)\cdot 100$$

We have noticed that the percent inhibition obtained using Eq. (2) would be generally slightly lower that that obtained using standard curve-based Eq. (1). Because a slight under-estimation of inhibition in fact makes the drug efficacy data more conservative, we hence used the simplified Eq. (2) in all calculations in this study.

### *In vitro* Screening of Natural Products

The NatProd Collection containing 800 pure chemicals of natural products were purchased from MicroSource Discovery Systems^1^ for discovering potential activities against the growth of *C. parvum in vitro* using qRT-PCR assay as described above. The primary screening was carried out for all 800 compounds at 10 μM containing 0.5% dimethyl sulfoxide (DMSO). In each 96-well plate, six wells were treated with 0.5% DMSO diluent as negative control, and two wells were treated with paromomycin at 150 μM as positive control. Secondary screening was conducted on compounds showing ≥60% inhibition on the parasite growth in primary screening at concentrations of 10 μM and 3.3 μM with the same experimental design as in the primary screening. Compounds showing ≥50% inhibition at 3.3 μM in the secondary screening were tested for dose-response curves to determine their EC_50_ values (half maximal effective concentration against the growth of *C. parvum in vitro*). All experiments included least two biological replicates for each compound in the *in vitro* drug treatment assay and two technical replicates for each biological replicate in the qRT-PCR assay. All primary and secondary screening assays were performed at least twice independently. For compounds showing disparities between replicates or experiments, the assays were repeated until data were convergent.

### Effect of Top Hits on Different Parasite Developmental Stages *in vitro* and Drug Withdrawal Assay

We selected three top hits derived from plants for evaluating their activity against various developmental stages of *C. parvum.* For evaluating the effect on the parasite invasion (i.e., 0–3 hpi treatment groups), host cell monolayers will be incubated with *C. parvum* oocysts (10^5^ oocysts/well) in 96-well plates together with individual compounds at around EC_80_ concentrations. For evaluating the effect on various stages of intracellular parasites (i.e., 3–10 hpi, 3–20 hpi, 22–44 hpi, and 3–44 hpi treatment groups), host cell monolayers were incubated with *C. parvum* oocysts (50,000 oocysts/well) for 3 h, followed by the removal of uninvaded parasites by a medium exchange. These time points roughly corresponded to the invasion into host cells (0–3 hpi), the development of first and some second generation of merogony (3–22 hpi) or second generation of merogony and gametogenesis (22–44 hpi). Individual compounds at EC_80_ final concentrations were added into wells at specified post-infection time points, and plates were incubated for specified durations of post-infection times. Cell lysates were prepared at the end of each treatment for qRT-PCR.

For drug withdrawal assay, HCT-8 cells were cultured and inoculated with *C. parvum* oocysts (50,000 oocysts/well) for 3 h. After a medium exchange, individual compounds at EC_80_ final concentrations were added into wells. Compounds were removed at 22 hpi time point by a medium exchange, and infected cells were allowed to grow up to 44 hpi (3–22 hpi treatment groups). A full course of treatment for each compound was included as control (3–44 hpi treatment groups). Cell lysates were prepared at 44 hpi time point for qRT-PCR.

In all experiments, negative controls were treated with diluent (0.5% DMSO) for the same durations of corresponding drug treatment groups. All experiments include least three biological replicates for each compound in the *in vitro* drug treatment assay and two technical replicates for each biological replicate in the qRT-PCR assay.

### *In vitro* Cytotoxicity Assay

Cytotoxicity of top hits on host cells was evaluated by an MTS assay (aka. one-step MTT assay) (Khabar et al., 1996). In this assay, HCT-8 cells were cultured in 96-well plates overnight, followed by incubation with compounds at serially diluted concentrations for 41 h. Plates were then rinsed 3 times with PBS, and incubated with a CellTiter 96 AQueous One Solution Cell Proliferation Assay (MTS) solution (20 μL/well) at 37°C for 90 min in a 5% CO_2_ incubator according to the manufacturer’s protocol (Promega, Madison, WI, United States). The plates were measured for absorbance at 490 nm in 15 min intervals in a SmartReader 96 microplate reader (Accuris, Edison, NJ, United States). Cytotoxicity of a compound was quantified by calculating the half maximal toxic concentration on host cells (TC_50_). An *in vitro* safety interval (SI) was calculated by determining the ratio between TC_50_ and EC_50_ values (i.e., SI = TC_50_/EC_50_).

## Results

### Phenotypic HTS Identified 16 Natural Products With Lower Micromolar to Sub-Micromolar Activity Against the Growth of *C. parvum in vitro*

**FIGURE 1:**
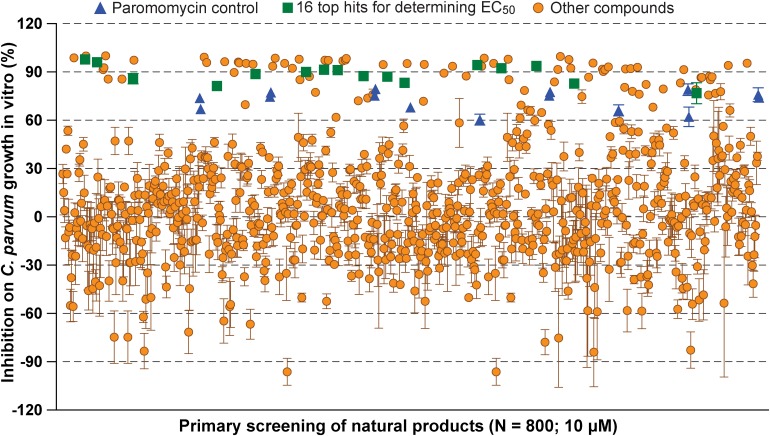
Scatter plot of the primary screening of 800 natural products (10 μM) against the growth of *Cryptosporidium parvum in vitro*. Blue triangles represent data from the positive control compound paromomycin (150 μM). Green squares represent the 16 top hits selected for determining their antiparasitic half maximal effective concentration (EC_50_) values and cytotoxicity shown in Table 1 and Figure 2. Each plate included 0.5% dimethyl sulfoxide diluent only as negative control (6 wells/plate). Bars show the standard error of the mean (*N* ≥ 3).

**FIGURE 2:**
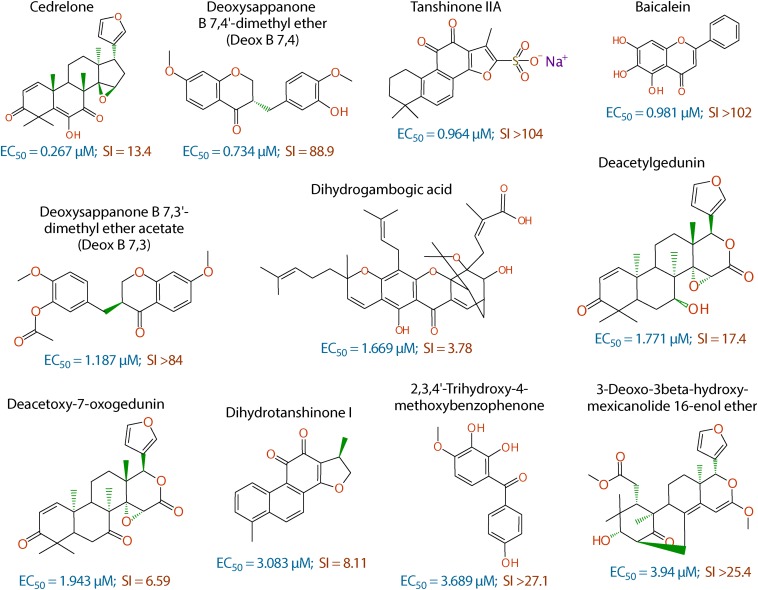
Chemical structures of the 11 top hits derived from plants and their *in vitro* EC_50_ and safety interval (SI) values.

In primary screening at 10 μM, we have observed a wide range of activity of the 800 natural products on the growth of *C. parvum in vitro* (i.e., ranging from −96.29% to 99.93% inhibitions) (Figure 1 and Supplementary Table S1). Based on Hs18S ΔC_T_ values between treatment and control groups, 20 compounds were highly cytotoxic to host cells at 10 μM and removed from the hit list. Among the remaining 780 compounds, we identified 88 compounds that inhibited the parasite growth by >60%. The 88 compounds were subjected to secondary screening at 10 and 3.3 μM, in which 29 compounds retained >50% inhibition activity at 3.3 μM (Supplementary Table S2). Subsequent dose-response experiments identified 16 compounds out of the 29 hits that exhibited lower to sub- micromolar EC_50_ values (i.e., EC_50_ < 4.0 μM) against the parasite growth *in vitro* (Figure 2 and Table 1), which represents 2% of the 800 natural products in the NatProd collection.

**TABLE 1:** *In vitro* anti-cryptosporidial activity, cytotoxicity, and safety interval (SI) of 16 top hits identified from 800 natural products^a^.

**Compound**	**Source (species)**	**CAS #**	**Description**	**EC_50_ (μM)**	**TC_50_ (μM)**	**SI^b^**
Valinomycin	Microbe (*Streptomyces*)	2001-95-8	Antibiotic; cyclic peptide ionophore	0.122	>50	>410
Mitomycin	Microbe (*Streptomyces*)	50-07-7	Antibiotic, Antineoplastic; DNA synthesis inhibitor	0.133	13.1	98.7
**Cedrelone**	**Plant (*Toona ciliate*)**	**1254-85-9**	**Antineoplastic**	**0.267**	**3.59**	**13.4**
Dactinomycin	Microbe (*Streptomyces*)	50-76-0	Antineoplastic; DNA-binding/RNA synthesis inhibitor	0.314	2.82	9.10
**Deoxysappanone B 7,4′- dimethyl ether (Deox B 7,4)**	**Plant (*Biancaea sappan*)**	**674786-37-9**	**Anti-leukemic; microtubule inhibitor**	**0.734**	**64.9**	**88.9**
Tanshinone IIA	Plant (Salvia miltiorrhiza)	568-72-9	Antineoplastic, bone resorption inhibitor, antiproliferative, apoptosis inducer, anti-inflammatory	0.964	>100	>104
**Baicalein**	**Plant (*Scutellaria*)**	**491-67-8**	**Antiviral (HIV), anti-inflammatory**	**0.981**	**>100**	**>102**
Deoxysappanone B 7,3′- dimethyl ether acetate	Plant (*Biancaea*)	356.3788	Human tyrosyl-DNA phosphodiesterase 1 inhibitor	1.187	>100	>84
Daunorubicin	Microbe (*Streptomyces*)	20830-81-3	Antibiotic, Antineoplastic	1.494	42.6	28.5
Dihydrogambogic acid	Plant (*Garcinia hanburyi*)		Matrix metalloproteinase 1 inhibitor	1.669	6.31	3.78
Deacetylgedunin	Plant (*Azadirachta indica*)	10314-90-6	Antiplasmodial, anti-inflammatory	1.771	30.9	17.4
Deacetoxy-7-oxogedunin	Plant (*Carapa guianensis*)	13072-74-7	Antiplasmodial activity	1.943	12.8	6.59
Lovastatin	Microbe (*Aspergillus terreus*)	75330-75-5	Antihyperlipidemic, HMGCoA reductase inhibitor	2.406	>100	>41.6
Dihydrotanshinone I	Plant (*Salvia miltiorrhiza*)	87205-99-0	Used for treating cardiovascular diseases	3.083	25.0	8.11
2,3,4′-Trihydroxy-4-methoxybenzophenone	Plant (*Anemarrhena asphodeloides*)	260.24874	Natural product derivative	3.689	>100	>27.1
3-Deoxo-3beta-hydroxy-mexicanolide 16-enol ether	Plant (unknown)	484.59492	Natural product derivative	3.940	>100	>25.4

*^*a*^Compounds in bold fonts were subjected to further study of their effects on various parasite developmental stages. ^*b*^SI, safety interval.*

The anti-cryptosporidial EC_50_ values of the 16 top hits ranged from 0.122 μM to 3.940 μM, in which 7 compounds had EC_50_ values <1.0 μM (Figure 2 and Table 1). Among the 7 top hits, three compounds were antibiotics derived from *Streptomyces* bacterial species, including valinomycin (#1 hit; EC_50_ = 0.122 μM), mitomycin (#2 hit; EC_50_ = 0.133 μM), and dactinomycin (#4 hit; EC_50_ = 0.314 μM), while four compounds were derived plants, including cedrelone (#3 hit; EC_50_ = 0.267 μM), deoxysappanone b 7,4′-dimethyl ether (Deox B 7,4) (#5 hit; EC_50_ = 0.734 μM), tanshinone IIA (#6; EC_50_ = 0.964 μM), and baicalein (#7; EC_50_ = 0.981 μM) (Table 1). The remaining 9 compounds with EC_50_ values ranging from 1.187 μM (deoxysappanone B 7,3′-dimethyl ether acetate) to 3.940 μM (3-deoxo-3beta-hydroxy-mexicanolide 16-enol ether) includes 7 derived from plants and two from microbes (Table 1).

All 16 top hits were efficacious on *C. parvum* at concentrations non-toxic to HCT-8 host cells, with TC_50_ ranging from 2.82 μM (dactinomycin) to >100 μM (6 compounds) (Figure 2 and Table 1). Twelve compounds had *in vitro* safety intervals (SIs) greater than 10 (i.e., SI = 13.4 to >410), while four compounds had lower SI values (i.e., SI = 4.52 to 9.10).

### Effect of Cedrelone, Deox B 7,4 and Baicalein on Various Parasite Developmental Stages

**FIGURE 3:**
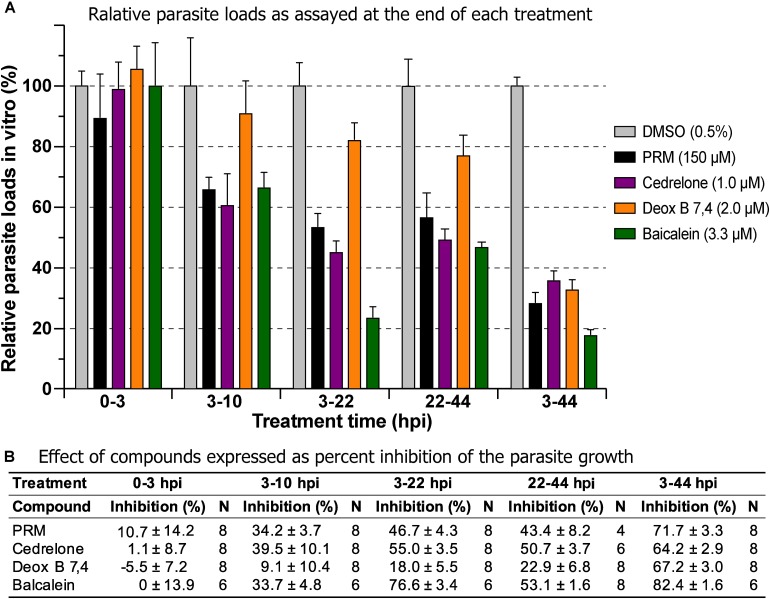
Effects of cedrelone (1.0 μM), Deox B 7,4 (deoxysappanone B 7,4′-dimethyl ether) (2.0 μM) and baicalein (3.3 μM) on various developmental stage of *Cryptosporidium parvum in vitro*. **(A)** The effects were expressed as relative parasite loads in bar chart. **(B)** The effects were shown as percentage inhibitions on the parasite growth. The data included the effects on the excystation and invasion of sporozoites (0-3 hpi treatment group), early developmental stages representing first generation and some second generation of merogony (3–10 hpi and 3–22 hpi), and second generation of merogony and gametogenesis stage (22–44 hpi). Intracellular parasites receiving a full course of treatment were used for comparison (3–44 hpi). Diluent [dimethyl sulfoxide (DMSO) at 0.5%] only was used as a negative control. Paromomycin (PRM; 150 μM) was used as a positive control. In this assay, parasite loads were determined at the end of each treatment. hpi, hours post-infection time. Bars show the standard error of the mean (*N* = 6 or 8).

Three of the top hits derived from plants were further evaluated for their effect on various developmental stages of *C. parvum* at around EC_60_ to EC_80_ concentrations (i.e., cedrelone at 1.0 μM, Deox B 7,4 at 2.0 μM, and baicalein at 3.3 μM). All three compounds had no effect on the invasion of *C. parvum* sporozoites into host cells (Figure 3; 0–3 hpi treatment group). For intracellular parasites after 3 hpi, the levels of inhibition by the three compounds and paromomycin (PRM) control were generally correlated with the treatment length (Figure 3). However, for cedrelone or baicalein, a shorter treatment time (3–22 hpi group) could achieve a level of inhibition comparable to that of a full course of treatment (3–44 hpi group) (Figure 3), implying a relatively quick action of the two natural products on *C. parvum in vitro*. There were no highly significant differences between 3 and 22 hpi (earlier development) and 22–44 hpi (relatively later development) treatment groups for the three compounds.

**FIGURE 4:**
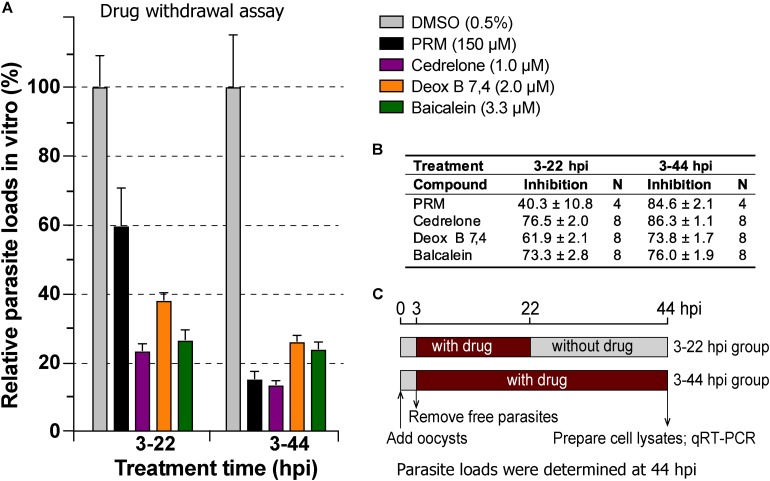
Drug withdrawal assay to evaluate the reversibility of the inhibition by cedrelone (1.0 μM), Deox B 7,4 (deoxysappanone B 7,4′-dimethyl ether) (2.0 μM), and baicalein (3.3 μM) on the growth of *C. parvum in vitro*. **(A)** Data were expressed as relative parasite loads in bar chart. **(B)** Data were shown as percentage inhibition on the parasite growth. **(C)** Illustration of the assay. In this assay, the parasites received treatments by individual compounds from 3 to 22 hpi time points, followed by the removal of compounds and continuous growth for up to 44 hpi time point. Intracellular parasites receiving a full course of treatment were used for comparison (3–44 hpi). Diluent (dimethyl sulfoxide at 0.5%) only was used as a negative control. Paromomycin (PRM; 150 μM) was used as a positive control. In this assay, parasite loads were determined at 44 hpi. hpi, hours post-infection time. Bars show the standard error of the mean (*N* = 4 or 8).

In drug withdrawal experiment suggested that the killing of the parasite by the three compounds were irreversible, because the parasites were unable to recover the growth well after being treated for 19 h (3–22 hpi treatment group) and allowed to growth without drugs for up to 44 hpi time point (vs. the 3–44 group receiving a full course of treatment) (Figure 4). It was also noticeable that Deox B 7,4 acted on intracellular *C. parvum* differently from the other two compounds. When intracellular parasites received 3–22 hpi treatment with Deox B 7,4, the parasite loads were high when assayed at 22 hpi (i.e., 18.0% inhibition vs. 67.2% inhibition in 3–44 group in Figure 3), but were unable to maintain growth after the removal of compound when assayed at 44 hpi (i.e., 61.9% inhibition vs. 73.8% inhibition in 3–44 group in Figure 4). These observations suggested that Deox B 7,4 treatment produced a “delayed death effect” of intracellular *C. parvum in vitro*.

## Discussion

The present study represents the first large scale screening of natural products for discovering novel anti-cryptosporidial activity *in vitro*. By phenotypic screening of 800 natural products with defined structures (Supplementary Tables S1, S2), we identified 16 top hits showing low to sub-micromolar levels of activity against the growth of *C. parvum in vitro* (anti-cryptosporidial EC_50_ values ranging from 0.122 to 3.940 μM). The 16 top hits represented compounds derived from microbes (*n* = 5) or plants (*n* = 11) (Figure 2 and Table 1). Among microbial-derived compounds, *in vitro* anti-cryptosporidial activity of mitomycin and lovastatin was previously reported (Armson et al., 1999; Bessoff et al., 2013; Ehrenman et al., 2013). Daunorubicin was identified as one of the top hits against *C. parvum in vitro* in a recent phenotypic HTS of marketed drugs by us (Guo et al., 2018), although it was found earlier to be ineffective on cryptosporidial infection in rats (Lemeteil et al., 1993). The activity of valinomycin and dactinomycin against *Cryptosporidium in vitro* was previously unreported.

The remaining 11 compounds were phytochemicals from various plant species, for which their novel anti-cryptosporidial activities were observed for the first time. The most efficacious compound was cedrelone (EC_50_ = 0.267 μM; SI = 13.4), which was a limonoid derived from the red cedar *Toona ciliate* or related species. Cedrelone was previously found to possess certain anti-microbial and *in vitro* anti-cancer activities (Liu et al., 2012; Fuzer et al., 2013; Wang et al., 2016), while no anti-parasitic activity was reported. The other three phytochemicals with sub-micromolar activity include: the homoisoflavanoid Deox B 7,4 (EC_50_ = 0.734 μM) from heartwood of *Biancaea sappan* (syn. *Caesalpinia sappan*) known for anti-leukemic and anti-microtubule activity (Bernard et al., 2015); tanshinone IIA (EC_50_ = 0.964 μM) as the main effective component of *Salvia miltiorrhiza* known as ‘Danshen’ in traditional Chinese medicine for treating cardiovascular disorders, as well as anti-inflammatory/antioxidant activities (Shang et al., 2012); and baicalein (EC_50_ = 0.981 μM) from the root of *Scutellaria baicalensis* and *S. lateriflora* known as one of the active ingredients of Xiaochaihutang or Sho-Saiko-To, a Chinese herbal supplement believed to enhance liver health, as well as anti-cancer and anti-*Leishmania* activities (Bosedasgupta et al., 2008; Gao et al., 2016; Liu et al., 2016).

Other phytochemicals were slightly less efficacious with anti-cryptosporidial EC_50_ values ranging from 1.187 to 3.940 μM. Among them, Deox B 7,3, an anolog of Deox B 7,4, was previously identified as one of the inhibitors of cardiomyocyte hypertrophy (Reid et al., 2016). Dihydrogambogic acid from a medical plant *Garcinia hanburyi* has been used topically to treat inflammatory skin disorders in China and found to be able to inhibit human matrix metalloproteinase 1 (Li et al., 2017). Deacetylgedunin is a limonoid (the 7-deacetyl derivative of gedunin) from *Azadirachta indica* known to possess anti-inflammatory and anti-malarial activities (Pereira et al., 2014; Sarigaputi et al., 2015; Akihisa et al., 2017; Chen et al., 2017). Deacetoxy-7-oxogedunin from *Carapa guianensis*, an analog of deacetylgedunin, was known for anti-malarial, hepatoprotective and collagen synthesis-promoting activities (Miranda Junior et al., 2012; Henriques and Penido, 2014; Pereira et al., 2014; Ninomiya et al., 2016; Morikawa et al., 2018). Dihydrotanshinone I is an analog of tanshinone IIA from *S. miltiorrhiza* with anti-cancer and anti-angiogenic activities (Zhang et al., 2012; Lee et al., 2017). The final two compounds, 2,3,4′-trihydroxy-4-methoxybenzophenone (PubChem CID: 3908719) and 3-deoxo-3beta-hydroxymexicanolide 16-enol ether (CID: 6708594), were derivatives of natural products, for which significant biological activities have yet been reported.

At this stage of investigation, we were focusing on plant-derived compounds. Our further investigation of three top phytochemicals (i.e., cedrelone, Deox B 7,4 and baicalein) indicated that a relatively short time of treatment of all three compounds produced long-term inhibition on the intracellular parasites (i.e., irreversible killing) (Figure 4). Among them, cedrelone and baicalein inhibited the parasite growth in a time-dependent manner, whereas Deox B 7,4 produced a “delayed death effect” on *C. parvum*. A “delayed death effect” was observed in other apicomplexan parasites when the replication of apicoplast organelles was inhibited (Pfefferkorn et al., 1992; Fichera et al., 1995; He et al., 2001; Rathore et al., 2010). In an earlier study on the anti-leukemic mechanism revealed that Deox B 7,4 was a reversible microtubule inhibitor that bound near the colchicine site, and could increase lysosomal V-ATPase activity and lysosome acidity in leukemia cells (Bernard et al., 2015). However, it still remains to be determined on whether the anti-cryptosporidial activity of Deox B 7,4 was attributed to its inhibition of the parasite microtubules. The Deox B 7,4-induced “delayed death effect” in *C. parvum* indicates that the pathway targeted by the compound is vital to the parasite, but it takes time to deplete its function after being inhibited by Deox B 7,4.

The discovery of new bioactive chemical scaffolds provides an opportunity for structure-activity relationship (SAR) to develop more effective and selective anti-cryptosporidial compounds. Using the top hit cedrelone as an example, although this compound is described as a derivative from the red cedar *T. ciliate*, its derivatives have been extracted from various plants, e.g., 9 and 10 cedrelone limonoids from *Walsura yunnanensis* and *Trichilia americana*, respectively (Ji et al., 2014, 2015). Using fingerprint Tanimoto-based 2-dimensional similarity search, we were also able to identify 215 compounds that shared ≥90% Tanimoto similarity with cedrelone from the PubChem database^2^. It is noticeable that 4 of the 11 phytochemical top hits identified from 800 structurally diverse natural products are cedrelone limonoids, including cedrelone, 3-deoxo-3beta-hydroxymexicanolide 16-enol ether, deacetoxy-7-oxogedunin, and deacetylgedunin with structure similarity scores between 0.82792 and 0.83495 (vs. cedrelone) based on the Rubberbanding Forcefield similarity analysis implanted in DataWarrior version 5.0.0 (Sander et al., 2015). Therefore, there are opportunities to acquire a sufficient number of cedrelone derivatives for subsequent SAR analysis. Similarly, derivatives of other top hits are also available or extracted from plants in collaborating with experts in natural products for SAR analysis and discovery of new leads.

In summary, we have identified a significant number of natural products showing novel anti-cryptosporidial activities, in which detailed anti-parasitic efficacies *in vitro*, cytotoxicity and safety margins for 16 top hits were studied, together with more detailed analysis on the activity of three top phytochemicals. These findings, particularly the plant-derived natural products, provide us a large selection of new structures to be explored for developing anti-cryptosporidial therapeutics, such as further evaluating their efficacy in animal models, exploring their analogs for discovering more efficacious and safer inhibitors (hit-to-lead), and identifying their drug targets.

## Data Availability

All datasets generated for this study are included in the manuscript and/or the Supplementary Files.

## Author Contributions

GZ and HZ conceived and designed the study. ZJ designed and conducted the experiments. JM assisted in conducting the experiments. ZJ and HZ conducted the data analysis. GZ, HZ, and ZJ wrote the manuscript. All authors contributed to the manuscript revision, read and approved the submitted version.

## Conflict of Interest Statement

The authors declare that the research was conducted in the absence of any commercial or financial relationships that could be construed as a potential conflict of interest.

## Footnotes

1 http://www.msdiscovery.com

2 https://pubchem.ncbi.nlm.nih.gov
