# Supplementary material for: Discovery of Novel Anti-cryptosporidial Activities From Natural Products by in vitro High-Throughput Phenotypic Screening
Source: Front Microbiol. 2019 Aug 29;10:1999. doi: 10.3389/fmicb.2019.01999 (PMC6736568; doi:10.3389/fmicb.2019.01999)
Supplement: Supplementary file 1 [file Table_1.pdf]

Table S1. Primary screening at 10 uM: Anti-cryptosporidial activity shown as percent inhibition and cytotoxicity as evaluated by host cell Hs18S ΔCT values between treated and control groups. N/A: not applicable

#	Compound name	CAS #	Formula	Mol Wt	Bioactivity and note	Plate #	Inhibition (%)	SEM	N	Hs18S ΔC_T	SEM	N	Cytotoxicity (%)
1	DIGOXIN	20830-75-5	C41H64O14	780.96	cardiac stimulant	p1 4-4	NA	NA	NA	14.32	0.10	2	100.0
2	DIGITOXIN	71-63-6	C41H64O13	764.96	inotropic, cardiotonic	p10 4-1	NA	NA	NA	13.47	0.04	4	100.0
3	PROSCILLARIDIN	466-06-8	C29H40O9	532.64	cardiotonic	p10 3-8	NA	NA	NA	13.28	0.08	4	100.0
4	STROPHANTHIDIN	66-28-4	C23H32O6	404.51	cardiotonic	p8 6-6	NA	NA	NA	11.98	0.27	4	100.0
5	OUABAIN	11018-89-6, 630-60-4 [anhydrous]	C29H44O12	584.67	antiarrhythmic, cardiotonic, hypertensive, Na/K ATPase inhibitor	p10 9-4	NA	NA	NA	11.65	0.21	4	100.0
6	DIHYDROCELASTRYL DIACETATE	0	C33H44O6	536.72	chaperone stimulant	p3 6-4	NA	NA	NA	11.29	0.08	4	100.0
7	CONVALLATOXIN	508-75-8	C29H42O10	550.65	cardiotonic	p5 1-8	NA	NA	NA	10.84	0.08	4	99.9
8	GAMBOGIC ACID	2752-65-0	C38H44O8	628.77	antiinflammatory, cytotoxic, inhibits HeLa cells in vitro;	p4 8-7	NA	NA	NA	10.02	0.76	4	99.9
9	PERUVOSIDE	1182-67-2	C30H44O9	548.68	cardiotonic	p1 7-3	NA	NA	NA	10.85	0.13	4	99.9
10	LANATOSIDE C	17575-22-3	C49H76O20	985.14	cardiotonic	p8 2-2	NA	NA	NA	8.93	0.41	4	99.8
11	GITOXIGENIN DIACETATE	5996-03-2	C27H38O7	474.60		p4 9-3	NA	NA	NA	8.90	0.15	4	99.8
12	CANTHARIDIN	56-25-7	C10H12O4	196.20	irritant	p8 10-7	NA	NA	NA	7.39	0.40	4	99.4
13	3-OXOURSAN (28-13)OLIDE	0	C30H44O3	452.68		p7 1-5	NA	NA	NA	6.62	0.62	4	99.0
14	ANTHOTHECOL	10410-83-0	C28H32O7	480.56		p4 1-5	NA	NA	NA	6.51	0.62	4	98.9
15	POMIFERIN	572-03-2	C25H24O6	420.47	antioxidant	p3 1-4	NA	NA	NA	6.42	0.06	4	98.8
16	PODOFILOX	518-28-5	C22H22O8	414.42	antineoplastic, inhibits microtubule assembly, and human DNA topoisomerase II; antimitotic agent	p7 5-1	NA	NA	NA	5.42	0.10	4	97.7
17	7-DESACETOXY-6,7-DEHYDROGEDUNIN	0	C26H30O5	422.53		p4 5-1	NA	NA	NA	5.31	0.10	4	97.5
18	alpha-MANGOSTIN	6147-11-1	C24H26O6	410.47		p1 2-6	NA	NA	NA	4.36	0.14	4	95.1
19	CELASTROL	34157-83-0	C29H38O4	450.62	antineoplastic, NO synthesis inhibitor, chaperone stimulant	p2 5-2	NA	NA	NA	3.82	0.51	4	92.9
20	1,4,5,8-TETRAHYDROXY-2,6-DIMETHYLANTHROQUINONE	19079-10-8	C16H12O6	300.27		p8 9-2	NA	NA	NA	3.65	1.94	4	92.0
21	ESTRAGOLE	140-67-0	C10H12O	148.21	insect atttractant, skin irritant, carcinogen	p8 2-4	99.57	0.10	4	-0.22	0.09	4	-16.6
22	CEDRELONE	1254-85-9	C26H30O5	422.53		p1 6-1	99.50	0.02	4	1.38	0.14	4	61.7
23	DIHYDROGAMBOGIC ACID	0	C38H46O8	630.79		p1 4-3	97.74	0.05	4	-0.60	0.38	4	-52.0
24	ABAMECTIN (avermectin B1a shown)	71751-41-2	C48H72O14	873.10	antiparisitic	p8 3-6	97.65	0.69	4	0.59	0.03	4	33.4
25	AVERMECTIN A1a	0	C49H74O14	887.13	antiparasitic	p5 1-7	97.32	0.18	4	0.02	0.06	4	1.6
26	EMETINE DIHYDROCHLORIDE	316-42-7, 483-18-1	C29H42Cl2N2O4	553.58	inhibits RNA, DNA and protein synthesis	p2 1-4	97.26	0.12	4	1.41	0.13	4	62.4
27	ISOOSAJIN	5745-54-0	C25H24O5	404.47		p5 1-4	96.83	0.09	4	-0.24	0.04	4	-18.5
28	STROPHANTHIDINIC ACID LACTONE ACETATE	0	C25H32O7	444.53		p3 4-4	96.28	0.11	4	2.37	0.08	4	80.7
29	HEXAMETHYLQUERCETAGETIN	1251-84-9	C21H22O8	402.40		p3 1-7	95.76	0.38	4	1.01	1.12	4	50.3
30	DIGOXIGENIN	1672-46-4	C23H34O5	390.52		p3 6-5	95.61	0.22	4	0.33	0.15	4	19.2
31	TRYPTAMINE	61-54-1	C10H12N2	160.22	psychotropic	p8 7-2	95.48	0.27	4	-0.43	0.14	4	-34.7
32	CAPREOMYCIN SULFATE	1405-37-4, 11003-38-6 [capreomycin]	C25H46N14O12S	766.80	antibacterial, tuberculostatic	p7 2-8	95.26	0.39	4	0.30	0.07	4	18.5
33	CRYPTOTANSHINONE	35825-57-1	C19H20O3	296.37	inhibits angiogenesis	p4 2-8	95.26	0.39	4	0.18	0.07	4	11.9
34	ACETYL ISOGAMBOGIC ACID	0	C40H46O9	670.81		p3 7-5	95.25	0.23	4	1.57	0.05	4	66.4
35	PLUMBAGIN	481-42-5	C11H8O3	188.18	antibacterial, antifungal, tuberculostatic; antifeedant (worm)	p5 10-5	95.14	0.51	4	1.26	0.09	4	58.1
36	PRISTIMERIN	1258-84-0	C30H40O4	464.65	antineoplastic, antiinflammatory	p3 8-5	94.70	0.31	4	0.55	0.06	4	31.6
37	ERYTHROMYCIN	114-07-8	C37H67NO13	733.95	antibacterial	p6 2-8	94.62	0.66	4	0.62	0.11	4	35.0
38	NOBILETIN	478-01-3	C21H22O8	402.40	matrix metaloproteinase inhibitor; antineoplastic	p5 7-1	94.56	0.34	4	0.26	0.06	4	16.2
39	DACTINOMYCIN	50-76-0	C62H86N12O16	1255.45	antineoplastic, intercalating agent	p6 10-6	94.27	0.68	4	1.50	0.04	4	64.6
40	MONENSIN SODIUM (monensin A is shown)	22373-78-0, 17090-79-8 (monensin)	C37H63NaO10	690.90	antibacterial	p10 6-6	94.05	0.57	4	0.25	0.09	4	15.7
41	MITOMYCIN	50-07-7	C15H18N4O5	334.33	antineoplastic	p7 9-2	93.73	0.54	4	1.34	0.04	4	60.5

42	SILIBININ	22888-70-6	C25H22O10	482.45	hepatoprotective agent, antioxidant	p7 1-2	93.53	0.62	4	-0.02	0.14	4	-1.4
43	RESVERATROL	501-36-0	C14H12O3	228.25	antifungal, antibacterial	p4 1-2	93.53	0.62	4	-0.13	0.14	4	-9.6
44	DIMETHYL GAMBOGINATE	0	C40H49ClO8	693.28		p6 7-1	93.45	0.17	4	2.30	0.13	4	79.7
45	HAEMATOPORPHYRIN	14459-29-1	C34H38N4O6	598.71	antidepressant, antineoplastic	p8 9-4	93.06	0.57	4	1.45	1.28	4	63.3
46	TOMATINE	86273-92-9	C47H79NO21	994.15	antifungal, antibacterial, antiinflammatory agent	p9 3-6	92.89	0.17	4	0.04	0.04	4	2.7
47	DIHYDROCELASTROL	0	C29H40O4	452.64		p1 7-1	92.50	2.01	4	1.27	0.35	4	58.7
48	3-HYDROXYFLAVONE	577-85-5	C15H10O3	238.25		p8 3-8	92.36	1.08	4	-0.04	0.07	4	-2.7
49	VALINOMYCIN	2001-95-8	C54H90N6O18	1111.35	antibiotic; LD5 (rat, po) 4 mg/kg	p7 4-1	92.27	0.83	4	-0.13	0.06	4	-9.2
50	3alpha-ACETOXYDIHYDRODEOXYGEDUNIN	0	C30H40O7	512.65		p4 4-1	92.27	0.83	4	-0.24	0.06	4	-18.1
51	RUTILANTINONE	21288-61-9	C22H20O9	428.40	coccidiostat	p9 2-6	91.98	0.24	4	-0.54	0.07	4	-45.2
52	BENZYL ISOTHIOCYANATE	622-78-6	C8H7NS	149.22	antineoplastic, antibacterial, antifungal	p9 1-8	91.81	0.55	4	0.57	0.09	4	32.8
53	beta-SITOSTEROL	83-46-5	C29H50O	414.72		p8 8-5	91.74	0.45	4	0.43	0.20	4	25.8
54	URIDINE TRIPHOSPHATE TRISODIUM	19817-92-6	C9H12N2Na3O15P3	550.09	psychostimulant	p8 2-1	91.48	2.02	4	0.23	0.14	4	14.5
55	DEMETHYLNOBILETIN	2174-59-6	C20H20O8	388.38		p1 6-8	91.41	1.46	4	-0.06	0.08	4	-4.6
56	PATULIN	149-29-1	C7H6O4	154.12	antibacterial	p9 10-4	91.38	0.40	4	0.81	0.10	4	43.0
57	DEOXYSAPPANONE B 7,4'-DIMETHYL ETHER	0	C18H18O5	314.34		p4 8-6	91.32	0.59	4	0.62	0.05	4	35.1
58	LAPACHOL	84-79-7	C15H14O3	242.28	antineoplastic, antifungal	p8 8-8	91.29	0.83	4	-0.60	0.04	4	-52.0
59	DEACETYLGEDUNIN	0	C26H32O6	440.54		p4 10-6	91.08	0.43	4	0.23	0.06	4	14.8
60	CHRYSIN DIMETHYL ETHER	21392-57-4	C17H14O4	282.30		p8 8-6	90.28	0.34	4	-0.12	0.10	4	-8.6
61	3-DEOXO-3beta-HYDROXYMEXICANOLIDE 16-ENOL ETHER	0	C28H36O7	484.59		p4 6-1	89.96	0.72	4	-0.49	0.06	4	-40.1
62	8beta-HYDROXYCARAPIN, 3,8-HEMIACETAL	0	C27H32O8	484.55		p3 5-7	89.65	0.47	4	0.18	0.09	4	11.8
63	5-HYDROXY-2',4',7,8-TETRAMETHOXYFLAVONE	123316-61-0	C19H18O7	358.35		p6 5-7	89.23	0.31	4	0.20	0.05	4	13.2
64	DEACETOXY-7-OXOGEDUNIN	0	C26H30O6	438.53		p3 8-8	88.69	1.41	4	0.39	0.09	4	23.6
65	3,16-DIDEOXYMEXICANOLIDE-3beta-DIOL	0	C27H36O7	472.58		p6 9-1	87.47	0.79	4	-0.18	0.02	4	-13.4
66	DEOXYSAPPANONE B 7,3'-DIMETHYL ETHER ACETATE	0	C20H20O6	356.38		p5 4-4	87.42	0.76	4	0.24	0.13	4	15.5
67	OBTUSAQUINONE	21105-15-7	C16H14O3	254.29		p4 6-5	87.13	1.25	4	0.84	0.05	4	44.3
68	DIHYDROTANSHINONE I	0	C18H14O3	278.31		p5 7-6	87.00	0.17	4	0.00	0.09	4	0.2
69	RESERPINE	50-55-5	C33H40N2O9	608.69	antihypertensive	p10 4-3	86.95	0.91	4	0.50	0.05	2	29.4
70	ASARYLALDEHYDE	4460-86-0	C10H12O4	196.20	fly attractant	p8 6-5	86.65	1.11	4	-0.29	0.31	4	-22.0
71	PACLITAXEL	33069-62-4	C47H51NO14	853.93	antineoplastic	p10 2-7	86.59	0.96	4	0.65	0.10	4	36.1
72	CYCLOSPORINE	59865-13-3	C62H111N11O12	1202.64	immunosuppressant	p10 2-6	86.52	0.40	4	0.16	0.04	4	10.4
73	LOVASTATIN	75330-75-5	C24H36O5	404.55	antihyperlipidemic, HMGCoA reductase inhibitor	p2 1-3	85.78	3.31	4	0.60	0.17	4	34.1
74	3-HYDROXYTYRAMINE	62-31-7	C8H11NO2	153.18	dopaminergic	p1 9-6	85.53	0.92	4	0.76	0.23	4	41.1
75	TRYPTOPHAN	73-22-3 ['L']	C11H12N2O2	204.23	antidepressant, nutrient; LD5(rat) 1634 mg/kg ip	p1 7-6	85.53	0.92	4	-0.75	0.11	3	-68.2
76	OCTOPAMINE HYDROCHLORIDE	104-14-3	C8H12ClNO2	189.64	adrenergic agonist	p7 5-2	85.13	0.39	4	-0.21	0.03	4	-15.8
77	BUSSEIN	41060-14-4	C43H54O18	858.90		p4 5-2	85.13	0.39	4	-0.33	0.03	4	-25.3
78	CYCLOVERATRYLENE	0	C27H30O6	450.54		p8 1-5	83.81	2.02	4	-0.03	0.07	4	-2.2
79	2,3,4'-TRIHYDROXY-4-METHOXYBENZOPHENONE	0	C14H12O5	260.25		p5 10-2	83.20	1.28	4	0.10	0.09	4	6.4
80	4-NONYLPHENOL	104-40-5	C15H24O	220.36	weevil pheromone, shows estrogenic activity	p9 9-8	83.12	1.61	4	0.14	0.09	4	9.2
81	TANSHINONE IIA	568-72-9	C19H18O3	294.35	antineoplastic, bone resorption inhibitor, antiproliferative, apoptosis inducer	p8 4-4	82.67	2.04	4	0.08	0.10	4	5.6
82	HARMINE	442-51-3	C13H12N2O	212.25	antiparkinsonian, CNS stimulant	p9 4-8	82.22	1.87	4	0.49	0.07	4	28.6
83	BAICALEIN	491-67-8	C15H10O5	270.24	antiviral (HIV)	p3 3-3	81.26	1.15	4	0.10	0.07	4	6.7

84	TETRANDRINE	518-34-3	C38H42N2O6	622.77	analgesic, antineoplastic, antihypertensive, lymphotoxin	p9 1-7	80.44	0.57	4	-0.04	0.02	4	-2.5
85	PICROPODOPHYLLIN	477-47-4	C22H22O8	414.42	Insulin growth factor 1 receptor inhibitor, antineoplastic	p9 7-4	79.38	1.02	4	0.94	0.15	4	47.8
86	IVERMECTIN	70288-86-7	C48H74O14	875.12	antiparasitic	p10 1-8	78.97	1.16	4	-0.05	0.08	4	-3.4
87	QUERCETIN	117-39-5, 6151-25-3(hydrate)	C15H10O7	302.24	capillary protectant, antioxidant. antineoplastic, anti-HIV	p7 7-4	78.42	1.12	4	0.34	0.03	4	21.1
88	TETRAHYDROGAMBOGIC ACID	0	C38H48O8	632.80		p9 2-8	77.89	0.85	4	-0.31	0.12	4	-23.6
89	CHENODIOL	474-25-9	C24H40O4	392.58	anticholithogenic, antilipemic agent	p10 5-5	77.73	5.03	4	0.27	0.09	2	17.2
90	3beta-HYDROXYDEOXODIHYDRODEOXYGEDUNIN	0	C28H38O6	470.61		p4 7-4	77.26	1.07	4	-0.26	0.11	4	-19.7
91	DAUNORUBICIN	20830-81-3	C27H29NO10	527.53	antineoplastic	p10 2-1	76.78	6.50	4	0.99	0.18	4	49.5
92	SIROLIMUS	53123-88-9	C51H79NO13	914.20	immunosuppressant, antineoplastic; rapamycin	p10 4-7	76.64	1.57	4	0.94	0.09	4	47.9
93	SAFROLE	94-59-7	C10H10O2	162.19	anesthetic (topical) and antiseptic, pediculicide	p9 3-8	76.16	2.02	4	-0.44	0.12	4	-35.3
94	CAMPTOTHECIN	7689-03-4	C20H16N2O4	348.36	antineoplastic	p5 5-7	75.97	3.37	4	1.35	0.01	4	60.8
95	TACROLIMUS	109581-93-3, 104987-11-3 [anhydrous]	C44H69NO12	804.04	immune suppressant, antifungal	p10 3-7	75.57	1.18	4	0.16	0.03	4	10.4
96	ROTENONE	83-79-4	C23H22O6	394.43	acaricide, ectoparasiticide, antineoplastic, mitochondrial poison	p8 5-5	74.65	4.19	4	1.24	0.05	4	57.8
97	PIPLARTINE	20069-09-4	C17H19NO5	317.34	anti-asthma, antibronchitis	p5 5-1	73.69	1.52	4	0.33	0.09	4	20.5
98	PODOPHYLLIN ACETATE	1180-34-3	C24H24O9	456.45		p5 3-6	72.02	0.99	4	0.58	0.16	4	33.2
99	VINBLASTINE SULFATE	143-67-9, 865-21-4	C46H60N4O13S	909.07	antineoplastic, spindle poison	p6 2-7	71.70	1.71	4	0.81	0.14	4	43.0
100	1,3-DIDEACETYL-7-DEACETOXY-7-OXOKHIVORIN	0	C26H34O7	458.56		p3 7-4	69.64	1.76	4	0.07	0.10	4	4.7
101	QUINIC ACID	77-95-2	C7H12O6	192.17		p7 6-8	69.36	1.31	4	0.23	0.06	4	14.6
102	GLUTATHIONE	70-18-8	C10H17N3O6S	307.33	antioxidant	p9 5-8	68.08	1.20	4	-0.40	0.05	4	-32.0
103	HESPERIDIN	520-26-3	C28H34O15	610.57	capillary protectant	p7 7-8	66.25	1.79	4	0.35	0.05	4	21.5
104	GRISEOFULVIN	126-07-8	C17H17ClO6	352.77	antifungal, inhibits mitosis in metaphase	p10 6-8	66.05	4.17	4	0.30	0.06	4	18.7
105	DIMETHYLSULFONE	67-71-0	C2H6O2S	94.13	antiinflammatory, antiproliferative, antiparasitic	p7 8-3	65.78	2.69	4	0.90	0.10	4	46.5
106	STIGMASTA-4,22-DIEN-3-ONE	20817-72-5	C29H46O	410.69		p7 9-8	64.69	2.24	4	0.46	0.06	4	27.4
107	MUNDULONE	481-94-7	C26H26O6	434.49		p4 10-4	61.78	2.00	4	0.22	0.06	4	14.4
108	DERRUSTONE	2204-59-3	C18H14O6	326.31		p7 6-7	61.29	2.74	4	0.39	0.05	4	23.9
109	LARIXINIC ACID	118-71-8	C6H6O3	126.11		p7 8-8	59.78	3.35	4	0.20	0.11	4	12.9
110	URSOCHOLANIC ACID	546-18-9	C24H40O2	360.59		p9 1-1	59.28	3.21	4	-0.10	0.02	4	-6.9
111	DESACETYL (7)KHIVORINIC ACID, METHYL ESTER	0	C28H40O10	536.63		p8 10-5	58.59	1.85	4	0.00	0.51	4	-0.1
112	CIMICIFUGOSIDE H1	163046-73-9	C35H52O9	616.80	estrogen	p6 8-1	58.34	15.15	4	1.71	1.07	4	69.4
113	ERYTHROSE	583-50-6	C4H8O4	120.11		p7 10-8	57.20	0.97	4	0.23	0.09	4	14.6
114	1,2alpha-EPOXYDEACETOXYDIHYDROGEDUNIN	0	C26H32O7	456.54		p5 10-1	56.48	4.23	4	-0.18	0.06	4	-12.9
115	PAPAVERINE HYDROCHLORIDE	61-25-6, 58-74-2	C20H22ClNO4	375.86	muscle relaxant (smooth), cerebral vasodilator	p10 5-8	56.16	1.09	4	0.57	0.08	4	32.7
116	D-(+)-MALTOSE	69-79-4	C12H22O11	342.30	nutrient, sweetener	p9 7-8	55.12	2.12	4	-0.19	0.06	4	-14.3
117	RHOIFOLIN	17306-46-6	C27H30O14	578.53		p9 1-6	54.61	3.06	4	-0.26	0.04	4	-20.0
118	GARDENIN B	2798-20-1	C19H18O7	358.35		p8 1-1	53.61	5.56	4	0.25	0.15	4	16.0
119	HEDERAGENIN	465-99-6	C30H48O4	472.71		p1 1-7	53.42	2.88	4	-0.11	0.17	4	-7.7
120	BLEOMYCIN (bleomycin B2 shown)	9041-93-4, 11056-06-7	C58H94N20O26S4	1615.77	antineoplastic	p7 6-3	52.96	4.42	4	-0.22	0.12	4	-16.8
121	ISOLIQUIRITIGENIN	961-29-5	C15H12O4	256.26	aldose reductase inhibitor, antineoplastic, antiinflammatory	p9 2-7	52.92	4.21	4	-0.34	0.10	4	-26.4
122	CANTHAXANTHIN (euglenanone)	514-78-3	C40H52O2	564.86		p9 3-7	52.72	2.15	4	-0.26	0.05	4	-19.8
123	KHIVORIN	2524-38-1	C32H42O10	586.69		p4 9-6	52.35	3.04	4	0.12	0.10	4	8.0
124	EVERNINIC ACID	570-10-5	C9H10O4	182.18		p9 8-8	52.13	4.07	4	-0.34	0.06	4	-26.6
125	3-HYDROXY-4-(SUCCIN-2-YL)-CARYOLANE delta-LACTONE	0	C19H28O4	320.43		p7 7-7	51.45	0.73	4	0.49	0.04	4	28.8

126	CASANTHRANOL [cascaroside A shown]	8024-48-4	C21H22O10	434.40	laxative, antineoplastic	p10 4-5	50.17	22.08	4	0.24	0.05	2	15.4
127	BRUCINE	4845-99-2, 357-57-3	C23H26N2O4	394.47	central stimulant	p10 9-8	49.94	5.54	4	-0.11	0.04	4	-8.2
128	3,7-DIMETHOXYFLAVONE	20950-52-1	C17H14O4	282.30		p8 9-6	49.73	2.16	4	-0.21	0.13	4	-15.4
129	BIXIN	39937-23-0	C25H30O4	394.52		p7 4-8	49.32	14.99	4	0.60	0.08	4	34.2
130	7,8-DIHYDROXYFLAVONE	38183-03-8	C15H10O4	254.24	vascular protectant, antihaemorrhagic, tyr kinase B agonist	p4 4-8	49.32	14.99	4	0.49	0.08	4	28.8
131	ETOPOSIDE	33419-42-0	C29H32O13	588.57	antineoplastic	p7 7-3	48.52	0.42	4	0.40	0.06	4	24.1
132	18-AMINOABIETA-8,11,13-TRIENE SULFATE	0	C20H33NO4S	383.55		p9 6-7	47.65	5.55	4	-0.19	0.04	4	-13.9
133	NARINGENIN	480-41-1	C15H12O5	272.26	antiulcer, gibberellin antagonist	p9 6-1	47.57	4.37	4	-0.23	0.12	4	-17.3
134	KASUGAMYCIN HYDROCHLORIDE	19408-46-9, 6980-18-3	C14H26ClN3O9	415.83	antifungal	p4 6-8	47.53	3.66	4	-0.02	0.04	4	-1.2
135	SALSOLIDINE	493-48-1	C12H17NO2	207.27	antihypertensive	p1 10-6	47.06	8.52	4	0.32	0.06	4	20.0
136	BICUCULLINE (+)	485-49-4	C20H17NO6	367.36	GABAa antagonist	p1 8-6	47.06	8.52	4	-1.43	0.95	4	-169.4
137	2-HYDROXY-5 (6)EPOXY-TETRAHYDROCARYOPHYLLENE	0	C15H26O2	238.37		p3 2-8	46.23	2.96	4	-0.15	0.08	4	-10.9
138	AVOCATIN A	0	C38H70O8	654.98	antibacterial, antifungal	p7 5-6	46.03	5.16	4	0.00	0.04	4	-0.1
139	ENTANDROPHRAGMIN	11013-05-1	C43H56O17	844.92		p4 5-6	46.03	5.16	4	-0.12	0.04	4	-8.3
140	MUPIROCIN	12650-69-0	C26H44O9	500.64	antibacterial, antimycoplasmal, isoleucyl-tRNA synthetase inhibitor	p10 4-8	45.65	1.20	4	0.09	0.08	4	5.8
141	ASTAXANTHIN	71772-51-5	C40H52O4	596.86		p1 4-1	45.11	4.28	4	-0.43	0.18	4	-34.8
142	DEGUELIN(-)	522-17-8	C23H22O6	394.43	antineoplastic, antiviral, insecticide	p3 5-5	45.04	6.26	4	0.60	0.30	4	34.2
143	URIDINE	58-96-8	C9H12N2O6	244.21		p5 7-8	44.81	4.43	4	0.02	0.08	4	1.4
144	DEOXYADENOSINE	16373-93-6	C10H13N5O3	251.25		p9 6-8	44.55	6.78	4	-0.35	0.10	4	-27.3
145	4'-METHOXYFLAVONE	4143-74-2	C16H12O3	252.27		p9 6-3	44.41	2.12	4	-0.09	0.22	4	-6.3
146	MYCOPHENOLIC ACID	24280-93-1	C17H20O6	320.35	immune suppressant, antineoplastic, antiviral	p10 8-4	44.40	4.30	4	1.03	0.08	4	51.1
147	PIMPINELLIN	131-12-4	C13H10O5	246.22	GABA receptor antagonist, phototoxin	p7 7-6	43.64	4.24	4	0.21	0.09	4	13.3
148	AGELASINE (stereochemistry of diterpene unknown)	0	C26H40ClN5	458.10	cytotoxic, antineoplastic	p7 6-6	43.55	7.01	4	0.27	0.02	4	16.8
149	SINENSETIN	2306-27-6	C20H20O7	372.38		p3 9-6	42.77	3.36	4	0.39	0.05	4	23.6
150	APRAMYCIN SULFATE	65710-07-8	C21H41N5O11	539.59	antibacterial; LD5(iv) 28mg/kg(mouse)	p10 6-7	42.68	7.42	4	-0.04	0.09	4	-3.1
151	COTININE	486-56-6, 5695-98-	C10H12N2O	176.22	antidepressant	P2 9-8	42.64	5.41	4	0.41	0.09	4	24.7
152	CHOLIC ACID, METHYL ESTER	1448-36-8	C25H42O5	422.61		p1 1-5	42.06	2.30	4	-0.11	0.06	3	-7.7
153	LUPEOL	545-47-1	C30H50O	426.73	antineoplastic	p5 6-8	41.81	7.76	4	0.05	0.04	4	3.4
154	BATYL ALCOHOL	544-62-7	C21H44O3	344.58		p9 1-5	41.32	1.54	4	-0.57	0.09	4	-48.0
155	MITRAPHYLLINE	509-80-8	C21H24N2O4	368.44	antineoplastic	p5 9-8	41.31	4.01	4	0.17	0.16	4	11.1
156	CARNOSINE	305-84-0	C9H14N4O3	226.24		p4 8-8	41.12	2.69	4	0.08	0.13	4	5.5
157	SISOMICIN SULFATE	53179-09-2, 32385-11-8	C19H39N5O11S	545.61	antibacterial, binds to ribosomes	p10 5-3	41.06	26.87	4	-0.11	0.09	2	-7.8
158	BOVINOCIDIN	504-88-1	C3H5NO4	119.08	antineoplastic	p9 4-7	41.04	3.08	4	-0.32	0.04	4	-25.1
159	7-DEACETOXY-7-OXOKHIVORIN	15004-51-0	C30H38O9	542.63		p3 9-8	40.16	1.36	4	0.21	0.06	4	13.5
160	6-HYDROXYFLAVONE	6665-83-4	C15H10O3	238.25		p8 5-2	39.99	5.24	4	0.08	0.04	4	5.6
161	CRUSTECDYSONE	5289-74-7	C27H44O7	480.65	insect molting hormone	p9 5-7	39.83	0.40	4	-0.15	0.03	4	-10.6
162	6,7,2',3',4'-PENTAMETHOXYISOFLAVONE	33978-66-4	C20H20O7	372.38		p2 6-4	39.55	3.29	4	-0.38	0.31	4	-30.1
163	DAIDZEIN	486-66-8	C15H10O4	254.24	phytoestrogen	p5 7-7	39.20	4.26	4	0.01	0.18	4	0.7
164	PHENETHYLAMINE HYDROCHLORIDE	156-28-5, 64-04-0 (base)	C8H12ClN	157.64	CNS stimulant	p8 10-2	39.04	2.69	4	2.99	2.02	4	87.4
165	2,3-DIHYDROXY-4-METHOXY-4'-ETHOXYBENZOPHENONE	0	C16H16O5	288.30		p9 3-4	38.96	3.86	4	-0.12	0.07	4	-8.6
166	L-DEOXYALLIIN	21593-77-1	C6H11NO2S	161.22	antineoplastic	p9 2-5	38.80	7.86	4	-0.29	0.13	4	-21.9
167	ANDROGRAPHOLIDE	5508-58-7	C20H30O5	350.46	antineoplastic	p5 4-5	38.80	1.92	4	0.08	0.02	4	5.3
168	ACARBOSE	56180-94-0	C25H43NO18	645.62	alpha-glucosidase & saccharase inhibitor, antidiabetes, antihyperlipidaemia, antiobesity	p10 5-7	37.83	2.12	4	0.39	0.04	4	23.6
169	HUPERZINE A	102518-79-6	C15H18N2O	242.32	anticholinesterase, cognition enhancer	p3 4-8	37.59	7.61	4	0.19	0.15	4	12.4
170	TUBOCURARINE CHLORIDE	6989-98-6, 57-94-3 [anhydrous], 41354-45-4 [replaced], 57-95-4	C37H42Cl2N2O6	681.66	muscle relaxant (skeletal)	p10 10-8	37.53	2.58	4	-0.29	0.13	4	-22.2
171	GINKGOLIDE A	15291-75-5	C20H24O11	440.41	antibacterial	p8 2-8	37.48	9.03	4	0.10	0.14	4	6.6

172	ORSELLINIC ACID	480-64-8	C8H8O4	168.15		p3 1-1	37.47	3.49	4	-0.25	0.09	4	-19.3
173	ROSMARINIC ACID	537-15-5	C18H16O8	360.32	antiinflammatory, antithrombotic, antiplatelet, cytostatic, antiviral	p3 1-5	37.27	1.47	4	-0.04	0.23	4	-3.1
174	SALICIN	138-52-3	C13H18O7	286.28	analgesic, antipyretic	p10 5-6	37.08	2.97	4	0.58	0.15	4	32.9
175	BETULIN	473-98-3	C30H50O2	442.73		p8 9-8	36.86	6.79	4	-0.49	0.06	4	-40.4
176	UTILIN	31218-22-1	C41H52O17	816.86		p3 10-8	36.84	1.74	4	0.06	0.09	4	4.2
177	ALEURETIC ACID	533-87-9	C16H32O5	304.43		p3 1-6	36.82	6.74	4	0.24	0.05	4	15.2
178	NICOTINE BITARTRATE	65-31-6	C18H26N2O12	462.41	nicotinyl acetylchloline receptor agonist, ectoparasiticide	p10 7-8	36.71	2.16	4	-0.52	0.11	4	-43.3
179	CYTIDINE	65-46-3	C9H13N3O5	243.22		p3 1-8	36.68	3.67	4	-0.53	0.34	4	-44.6
180	DEOXYPEGANINE HYDROCHLORIDE	61939-05-7	C11H13ClN2	208.69	acetylcholinesterase inhibitor, antiParkinsonism	p5 6-5	36.29	9.69	4	-0.06	0.12	4	-4.6
181	HYOSCYAMINE	101-31-5	C17H23NO3	289.38	anticholinergic, analgesic	p10 5-2	36.17	29.26	4	-0.12	0.01	2	-8.9
182	BERGENIN	477-90-7	C14H16O9	328.28	hepatoprotectant	P2 9-6	36.01	9.98	4	0.38	0.18	4	23.1
183	GITOXIN	4562-36-1	C41H64O14	780.96	cardiotonic	p1 3-5	35.41	4.02	2	0.08	0.04	2	5.4
184	NYSTATIN	114-90-9	C47H75NO17	926.12	antifungal, binds to membrane sterols	p10 4-4	34.97	20.87	4	1.05	0.58	4	51.7
185	COLISTIN SULFATE	1264-72-8	C52H102N16O21S2	1351.62	antibacterial	p10 10-7	33.43	13.56	4	0.01	0.05	4	0.4
186	DIHYDROGEDUNIN	0	C28H36O7	484.59		p3 7-8	33.28	4.27	4	-0.24	0.04	4	-17.9
187	RETUSIN	1245-15-4	C19H18O7	358.35		p9 10-5	33.24	4.57	4	0.27	0.06	4	17.0
188	BETULINIC ACID	472-15-1	C29H46O3	442.69	antineoplastic	p9 5-6	32.95	4.45	4	-0.23	0.10	4	-17.4
189	DALBERGIONE	0	C15H12O2	224.26		p5 9-3	32.77	5.71	4	0.33	0.07	4	20.4
190	GIBBERELLIC ACID	77-06-5	C19H22O6	346.38		p3 5-8	32.70	3.99	4	0.09	0.13	4	5.9
191	MORIN	480-16-0	C15H10O7	302.24	P45 and ATPase inhibitor	p4 7-8	32.34	4.90	4	-0.21	0.04	4	-15.6
192	LACTOSE MONOHYDRATE	62-42-3	C12H24O12	360.32	nutrient, pharmaceutic aid	p4 6-6	31.71	8.06	4	0.02	0.04	4	1.4
193	MEXICANOLIDE	1915-67-9	C27H32O7	468.55		p3 6-7	31.47	8.07	4	-0.17	0.05	4	-12.3
194	MEPARTRICIN	11121-32-7	C60H88N2O19	1141.37	antifungal, antiprotozoal	p7 2-1	31.44	5.64	4	-0.51	0.08	4	-42.4
195	CARAPIN-8(9)-ENE	0	C27H30O7	466.54		p4 2-1	31.44	5.64	4	-0.62	0.08	4	-54.0
196	LARIXOL	0	C20H34O2	306.49		p2 5-8	31.41	4.95	4	0.03	0.12	5	2.2
197	DIHYDROGEDUNIC ACID, METHYL ESTER	0	C26H36O8	476.57		p4 6-4	31.36	5.53	4	-0.20	0.12	4	-15.2
198	SPAGLUMIC ACID	4910-46-7	C11H16N2O8	304.26	neurotransmitter; mGluR3 receptors	p5 10-8	31.28	6.91	4	0.18	0.24	4	11.9
199	ACTINONIN	13434-13-4	C19H35N3O5	385.51	antibacterial	p5 4-8	31.27	4.56	4	-0.06	0.04	4	-4.6
200	3-METHYLORSELLINIC ACID	4707-46-4	C9H10O4	182.18		p4 6-7	30.83	3.87	4	0.05	0.03	4	3.6
201	CARAPIN	3463-88-5	C27H32O7	468.55		p3 6-8	30.71	8.06	4	-0.07	0.10	4	-5.2
202	DALBERGIONE, 4-METHOXY-4'-HYDROXY-	0	C16H14O4	270.29		p5 4-2	30.34	5.31	4	0.09	0.07	4	6.1
203	PIPERONYLIC ACID	94-53-1	C8H6O4	166.13		p3 2-7	30.30	3.46	4	-0.04	0.10	4	-3.1
204	PACHYRRHIZIN	10091-01-7	C19H12O6	336.30	insecticide	p3 2-1	29.77	5.27	4	-0.08	0.10	4	-6.0
205	EPOXY (4,5alpha)-4,5-DIHYDROSANTONIN	0	C15H18O4	262.31		P2 8-8	29.68	13.61	4	-0.03	0.31	4	-1.8
206	PERILLYL ALCOHOL	536-59-4, 18457-55-1	C10H16O	152.24	antineoplastic, apoptosis inducer; skin irritant, LD5(rat) 21 mg/kg po	p5 2-5	29.51	1.43	4	-0.35	0.04	4	-27.4
207	QUASSIN	76-78-8	C22H28O6	388.46	insecticide, antiamoebic	p7 5-8	29.35	5.75	4	0.08	0.10	4	5.1
208	FERULIC ACID	1135-24-6	C10H10O4	194.19	antineoplastic, choleretic, food preservative	p4 5-8	29.35	5.75	4	-0.04	0.10	4	-2.6
209	KANAMYCIN A SULFATE	25389-94-0, 133-92-6 [replaced], 59-01-8	C18H38N4O15S	582.59	antibacterial	p10 6-2	29.26	9.55	4	-0.31	0.13	4	-23.9
210	EPOXYGEDUNIN	0	C28H34O8	498.58		p5 2-4	29.04	3.64	4	-0.46	0.08	4	-37.3
211	STRYCHNINE	57-24-9	C21H22N2O2	334.42	central stimulant	p10 8-8	29.02	2.64	4	-0.30	0.09	4	-23.5
212	PHLORIDZIN	60-81-1	C21H24O10	436.42	induces experimental glucosuria, antifeedant	p7 1-7	28.85	5.29	4	-0.08	0.02	4	-5.8
213	MENTHONE	14073-97-3	C10H18O	154.25		p4 1-7	28.85	5.29	4	-0.20	0.02	4	-14.5
214	PUTRESCINE DIHYDROCHLORIDE	0	C4H14Cl2N2	161.08	ornithine decarboxylase inhibitor, cell growth factor	p9 7-7	28.72	4.86	4	-0.04	0.11	4	-3.0
215	DEOXYGEDUNIN	21963-95-1	C28H34O6	466.58	neuroprotective, anti-depressant. learning enhancement	p2 5-1	28.62	9.62	4	0.24	0.09	4	15.4
216	6,4'-DIHYDROXYFLAVONE	63046-09-3	C15H10O4	254.24	antihaemorrhagic	p9 8-1	28.18	11.52	4	0.28	0.14	4	17.9
217	ESTRONE	53-16-7	C18H22O2	270.37	estrogen	p10 9-1	28.09	4.10	4	-0.24	0.05	4	-18.4

218	CADAVERINE TARTRATE	462-94-2(base)	C7H16N2O6	224.22		p4 9-8	27.52	5.85	4	-0.06	0.05	4	-4.5
219	DEOXYKHIVORIN	0	C32H42O9	570.69		p1 3-4	27.34	5.50	4	0.11	0.10	4	7.7
220	1,7-DIDEACETOXY-1,7-DIOXO-3-DEACETYLKHIVORIN	0	C26H32O7	456.54		p4 8-1	26.84	3.45	4	-0.10	0.04	4	-6.9
221	CHOLIC ACID	81-25-4	C24H40O5	408.58		p1 1-6	26.71	2.48	4	-0.40	0.03	4	-32.0
222	MEVALONIC ACID LACTONE	503-48-0	C6H10O3	130.14		p7 10-7	26.69	3.56	4	0.26	0.07	4	16.5
223	SCLAREOLIDE	564-20-5	C16H26O2	250.38	antimicrobial	p1 1-1	26.58	5.98	4	0.57	0.18	4	32.7
224	ADRENOLONE HYDROCHLORIDE	62-13-5, 99-45-6 (base)	C9H12ClNO3	217.65	adrenergic (opthalmic)	p6 8-8	26.43	5.66	4	-0.22	0.02	4	-16.6
225	LIMONIN	1180-71-8	C26H30O8	470.52		p7 1-8	26.18	5.78	4	-0.12	0.05	4	-8.4
226	HECOGENIN	467-55-0	C27H42O4	430.63	antiinflammatory	p4 1-8	26.18	5.78	4	-0.23	0.05	4	-17.3
227	LUPININE	486-70-4	C10H19NO	169.27	antifeedant, antiinflammatory, oxytoxic	p3 2-4	26.07	3.70	4	0.18	0.12	4	11.8
228	VERATRINE SULFATE	62-59-9	C32H51NO13S	689.83	antihypertensive	p6 10-8	25.98	3.40	4	-0.06	0.02	4	-4.3
229	LUPEOL ACETATE	1617-68-1	C32H52O2	468.77	antiulcer	p5 6-7	25.85	8.48	4	-0.17	0.06	4	-12.1
230	TYLOSIN TARTRATE	1405-54-5, 1401-69-	C50H83NO23	1066.21	antibacterial	p10 7-7	25.75	3.76	4	-0.19	0.10	4	-14.2
231	ALLYLISOTHIOCYANATE	57-06-7	C4H5NS	99.16	counterirritant	p10 5-4	25.73	5.30	4	0.28	0.05	4	17.7
232	THEAFLAVIN MONOGALLATES	0	C36H28O16	716.61		p3 7-7	25.59	5.29	4	-0.10	0.05	4	-7.4
233	SKATOLE	83-34-1	C9H9N	131.18	insect attractant	p7 8-7	25.24	4.65	4	0.30	0.04	4	18.5
234	ASIATIC ACID	464-92-6	C30H48O5	488.71	wound healing, experimental carcinogen	p5 6-1	25.12	1.68	4	-0.29	0.09	4	-22.7
235	COLCHICINE	64-86-8	C22H25NO6	399.45	antimitotic, antigout agent	p7 5-3	24.94	12.09	4	-0.34	0.08	4	-26.5
236	OLEANOIC ACID	508-02-1	C30H48O3	456.72		p4 5-3	24.94	12.09	4	-0.45	0.08	4	-36.8
237	COLFORSIN	66575-29-9	C22H34O7	410.51	adenylate cyclase activator, antiglaucoma, hypotensive, vasodilator	p10 1-7	24.79	7.28	4	0.14	0.05	4	9.4
238	CHRYSANTHEMIC ACID, ETHYL ESTER	0	C12H20O2	196.29	insecticide	p1 6-2	24.65	11.12	4	-0.97	0.06	4	-95.7
239	METAMECONINE	0	C10H10O4	194.19		p7 9-6	24.49	0.94	4	0.33	0.05	4	20.2
240	BISANHYDRORUTILANTINONE	749-18-8	C22H16O7	392.37	antibacterial	p9 7-6	24.47	2.89	4	-0.16	0.11	4	-12.0
241	DOCOSANOL	661-19-8	C22H46O	326.61	antiviral	p6 1-8	24.37	1.69	4	-0.38	0.06	4	-30.5
242	CURCUMIN	458-37-7	C21H20O6	368.39	antiedemic, antiinflammatory, bile stimulant; antibacterial, antifungal, lipo/cyclooxygenase inhibitor	p3 7-6	24.26	5.24	4	0.03	0.02	4	2.4
243	beta-CARYOPHYLLENE ALCOHOL	0	C15H26O	222.37		p3 3-8	24.19	6.28	4	0.03	0.04	4	1.9
244	MEVASTATIN	73573-88-3	C23H34O5	390.52	antihyperlipidemic, HMGCoA reductase inhibitor	p6 8-6	24.00	5.21	4	0.22	0.09	4	14.4
245	SPERMIDINE TRIHYDROCHLORIDE	0	C7H22Cl3N3	254.63	ornithine decarboxylase inhibitor	p8 7-1	23.90	2.28	4	-0.63	0.05	4	-54.4
246	3-NOR-3-OXOPANASINSAN-6-OL	0	C14H22O2	222.33		p2 1-8	23.88	9.87	4	0.24	0.07	4	15.3
247	2-METHYLENE-5-(2,5-DIOXOTETRAHYDROFURAN-3-YL)-6-OXO--10,10-DIMETHYLBICYCLO[7: 2: 0]UNDECANE	0	C18H24O4	304.39		p3 1-3	23.70	7.60	4	-0.09	0.11	4	-6.8
248	AZADIRACHTIN	11141-17-6	C35H44O16	720.73	antifeedant, insecticide	p7 1-6	23.68	7.08	4	0.00	0.03	4	-0.1
249	ORSELLINIC ACID, ETHYL ESTER	2524-37-0	C10H12O4	196.20		p4 1-6	23.68	7.08	4	-0.12	0.03	4	-8.3
250	METHYL GAMBOGATE METHYL ETHER	0	C40H48O8	656.82		P2 7-6	23.61	5.17	4	0.37	0.20	4	22.4
251	ADENOSINE PHOSPHATE	61-19-8	C10H14N5O7P	347.23	vasodilator, neuromodulator	p10 7-4	23.53	5.52	4	-0.16	0.02	4	-11.8
252	METHYL DEOXYCHOLATE	3245-38-3	C25H42O4	406.61		P2 10-7	23.38	14.27	4	0.66	0.07	4	36.7
253	DJENKOLIC ACID	498-59-9	C7H14N2O4S2	254.33		p3 3-1	23.33	8.14	4	0.00	0.05	4	0.2
254	HYPOXANTHINE	68-94-0	C5H4N4O	136.11		p3 9-7	23.21	5.53	4	0.22	0.13	4	14.3
255	PICROTOXININ	17617-45-7	C15H16O6	292.29	convulsant, GABA receptor antagonist, ichthyotoxin	p8 10-8	23.15	1.78	4	-0.81	0.36	4	-75.3
256	DANTHRON	117-10-2	C14H8O4	240.22	cathartic	p6 9-8	22.99	2.14	4	-0.12	0.05	4	-8.6
257	RAUWOLSCINE HYDROCHLORIDE	6211-32-1	C21H27ClN2O3	390.91	alpha2 adrenergic antagonist	p8 1-8	22.85	10.47	4	-0.17	0.05	4	-12.6
258	XANTHOPTERIN	119-44-8	C6H5N5O2	179.14	cell proliferation inhibitor	P2 10-2	22.72	5.69	4	0.47	0.20	4	27.9
259	2-ACETYLPYRROLE	1072-83-9	C6H7NO	109.13	hepatoprotectant, organoleptic	p9 2-4	22.62	11.65	4	-0.43	0.13	4	-34.6
260	DEHYDROVARIABILIN	0	C17H14O4	282.30		p2 4-5	22.03	3.24	4	-0.42	0.13	4	-33.6
261	L(+/-)-ALLIIN	556-27-4(-)	C6H11NO3S	177.22	antibacterial, antioxidant	p5 3-1	21.98	7.50	4	-0.17	0.05	4	-12.5
262	ESTRADIOL	50-28-2	C18H24O2	272.39	estrogen	p10 7-1	21.97	4.36	4	-0.19	0.07	4	-13.8
263	NALOXONE HYDROCHLORIDE	357-08-4, 51481-60-8 [dihydrate], 465-65-6	C19H22ClNO4	363.84	narcotic antagonist	p8 1-2	21.86	11.79	4	0.20	0.12	4	12.7

264	LACCAIC ACID A	15979-35-8	C26H19NO12	537.44		p3 2-6	21.81	2.10	4	-0.09	0.06	4	-6.6
265	GOSSYPOL	303-45-7	C30H30O8	518.57	antispermatogenic, antineoplastic, antiHIV	p9 7-5	21.38	3.16	4	0.45	0.05	4	26.6
266	HYDROCORTISONE	50-23-7	C21H30O5	362.47	glucocorticoid, antiinflammatory	p6 7-8	21.10	7.54	4	-0.47	0.07	4	-38.1
267	CHOLINE CHLORIDE	67-48-1, 62-49-7 [choline]	C5H14ClNO	139.63	choleretic, lipotropic, hepatoprotectant	p6 4-8	20.98	6.76	4	0.33	0.43	4	20.6
268	PICROPODOPHYLLIN ACETATE	0	C24H24O9	456.45		p8 8-1	20.82	8.69	4	1.02	0.05	4	50.6
269	FRIEDELIN	559-74-0	C30H50O	426.73		p4 6-3	20.81	6.77	4	-0.23	0.14	4	-17.6
270	CREATININE	60-27-5	C4H7N3O	113.12	metabolic enhancer	p5 6-6	20.47	12.29	4	0.00	0.03	4	0.2
271	THEOPHYLLINE	5967-84-0, 58-55-9	C7H8N4O2	180.17	bronchodilator	p6 3-8	20.47	4.96	4	-0.29	0.14	4	-22.6
272	CHRYSIN	480-40-0	C15H10O4	254.24	diuretic	p4 7-7	20.34	2.92	4	0.14	0.02	4	9.3
273	2-METHOXYRESORCINOL	29267-67-2	C7H8O3	140.14		p7 3-8	20.33	3.07	4	-0.20	0.09	4	-15.0
274	XANTHURENIC ACID	59-00-7	C10H7NO4	205.17	caspase activator, guanylyl cyclase stimulant	p4 3-8	20.33	3.07	4	-0.32	0.09	4	-24.4
275	DEOXYSAPPANONE B TRIMETHYL ETHER	0	C19H20O5	328.37		p5 3-4	20.25	5.01	4	0.04	0.07	4	2.4
276	4'-METHOXYCHALCONE	22966-19-4	C16H14O2	238.29		p2 4-4	20.03	0.97	4	-0.16	0.08	4	-12.0
277	BETAINE HYDROCHLORIDE	590-46-5, 141-58-2 [replaced], 107-43-7	C5H12ClNO2	153.61	antiarteriosclerotic, hypolipaemic, hepatoprotectant	p10 7-6	19.87	1.17	4	-0.38	0.05	4	-30.0
278	KAEMPFEROL	520-18-3	C15H10O6	286.24		p7 9-7	19.76	2.90	4	0.54	0.09	4	31.2
279	PAROMOMYCIN SULFATE	1263-89-4, 7542-37-2 [paromomycin], 59-04-1 [paromomycin, replaced]	C23H47N5O18S	713.72	antibacterial, antiamebic	p10 9-6	19.72	5.12	4	0.08	0.05	4	5.5
280	DIALLYL SULFIDE	592-88-1	C6H10S	114.21	antibacterial, antifungal, antineoplastic, antihypercholesterolaemic, hepatoprotectant	p8 1-3	19.62	2.17	4	-0.01	0.10	4	-0.8
281	3-AMINO-beta-PINENE	0	C10H18ClN	187.71		p2 3-6	19.24	13.24	4	0.42	0.15	4	25.3
282	EUPARIN	532-48-9	C13H12O3	216.24		p2 5-6	19.01	4.38	4	0.23	0.06	4	14.6
283	2,6-DIHYDROXY-4-METHOXYTOLUENE	0	C8H10O3	154.17		p9 7-1	18.96	8.38	4	0.06	0.12	4	4.4
284	PEONOL METHYL ETHER	829-20-9	C10H12O3	180.21		p9 4-6	18.92	6.94	4	-0.31	0.17	4	-24.3
285	CAFFEIC ACID	331-39-5	C9H8O4	180.16		p9 3-5	18.92	7.23	4	-0.34	0.10	4	-26.2
286	USNIC ACID	125-46-2	C18H16O7	344.32	antibacterial	p5 3-8	18.84	7.20	4	-0.31	0.05	4	-23.9
287	TANGERITIN	481-53-8	C20H20O7	372.38		p1 10-1	18.55	2.07	4	0.12	0.10	4	8.0
288	SANGUINARINE SULFATE	5578-73-4	C20H15NO8S	429.41	antineoplastic, antiplaque agent	p1 8-1	18.55	2.07	4	0.07	0.04	4	4.9
289	HELICIN	618-65-5	C13H16O7	284.27		P2 10-8	18.55	13.31	4	0.07	0.11	4	4.7
290	IZALPININ	480-14-4	C16H12O5	284.27		p8 5-1	18.39	12.07	4	0.18	0.14	4	11.5
291	LOBARIC ACID	0	C25H28O8	456.50		p3 2-5	18.17	5.37	4	0.20	0.12	4	13.1
292	DIHYDROSTREPTOMYCIN SULFATE	5490-27-7, 128-46-1 [dihydrostreptomycin]	C21H43N7O16S	681.68	antibacterial, tuberculostatic	p10 5-1	17.98	8.83	4	0.05	0.21	4	3.4
293	d,l-threo-3-HYDROXYASPARTIC ACID	4294-45-5	C4H7NO5	149.10	L-aspartate beta-carboxylase inhibitor	p1 7-8	17.79	2.80	4	0.16	0.04	4	10.7
294	HESPERETIN	520-33-2	C16H14O6	302.29		p7 6-5	17.64	6.87	4	-0.02	0.05	4	-1.2
295	OLEANDOMYCIN PHOSPHATE	3922-90-5 (base)	C35H64NO16P	785.87	antibacterial	p6 6-8	17.62	4.33	4	3.02	3.38	4	87.6
296	CATECHIN TETRAMETHYLETHER	0	C19H22O6	346.38		p2 4-3	17.53	5.71	4	-0.47	0.14	4	-38.4
297	CYANOCOBALAMIN	68-19-9	C63H92CoN14O14P	1359.43	vitamin, coenzyme B12	p10 7-5	16.98	4.68	4	-0.32	0.02	4	-24.5
298	CEPHARANTHINE	481-49-2	C37H38N2O6	606.73	antineoplastic, hepatoprotectant, radioprepective	p6 9-6	16.84	14.23	4	0.33	0.09	4	20.5
299	ARTEMISININ	63968-64-9	C15H22O5	282.34	antimalarial	p5 8-8	16.73	3.78	4	0.00	0.04	4	0.0
300	SALVINORIN A	83729-01-5	C23H28O8	432.47	k-opiod receptor agonist, psychotropic	p7 3-5	16.71	5.50	4	0.53	0.09	4	30.5
301	LEOIDIN	105350-54-7	C18H14Cl2O7	413.21		p4 3-5	16.71	5.50	4	0.41	0.09	4	24.9
302	EUCALYPTOL	470-82-6	C10H18O	154.25	anthelminthic, antiseptic, expectorant	p10 9-7	16.23	6.83	4	-0.22	0.04	4	-16.4
303	2-BENZOYL-5-METHOXYBENZOQUINONE	0	C14H10O4	242.23		p5 1-1	16.19	4.32	4	-0.59	0.02	4	-50.7
304	ARBUTIN	497-76-7	C12H16O7	272.26		P2 9-3	16.10	4.42	4	0.01	0.09	4	0.5
305	KAWAIN	3155-48-4	C14H14O3	230.27		p7 1-4	15.98	3.82	4	-0.15	0.07	4	-10.9
306	CYTISINE	485-35-8	C11H14N2O	190.25	antiinflammatory, respiratory stimulant	p4 1-4	15.98	3.82	4	-0.26	0.07	4	-20.0
307	PICEID METHYL ETHER	30197-14-9	C21H24O8	404.42	antioxidant	p8 10-6	15.82	7.55	4	0.52	0.33	4	30.3
308	AVOCADYNE ACETATE	24607-06-5	C19H34O4	326.48	antifungal	p5 8-7	15.78	4.31	4	0.19	0.08	4	12.2
309	4-HYDROXY-6-METHYLPYRAN-2-ONE	675-10-5	C6H6O3	126.11		p3 10-7	15.41	3.25	4	0.04	0.08	4	3.1
310	TIGOGENIN	77-60-1	C27H44O3	416.65		p2 4-7	15.39	2.37	4	-0.40	0.02	4	-31.8
311	STREPTOMYCIN SULFATE	3810-74-0, 57-92-1	C21H41N7O16S	679.66	antibacterial (tuberculostatic)	p10 7-3	15.36	5.64	4	-0.10	0.04	4	-7.1

312	HAEMATOMMIC ACID, ETHYL ESTER	39503-14-5	C11H12O5	224.22		P2 7-5	15.27	9.76	4	0.12	0.11	4	8.0
313	SOLANESYL ACETATE	0	C47H76O2	673.13		p4 6-2	15.24	4.90	4	-0.20	0.10	4	-14.8
314	ANDROSTA-1,4-DIEN-3,17-DIONE	897-06-3	C19H24O2	284.40		p3 6-6	15.07	12.25	4	0.01	0.11	4	0.7
315	VISNAGIN	82-57-5	C13H10O4	230.22		p1 1-2	15.05	13.87	3	-0.27	0.17	4	-20.7
316	2',4'-DIHYDROXY-4-METHOXYCHALCONE	81674-91-1	C16H14O4	270.29		p1 4-2	14.88	10.99	4	-1.00	0.20	4	-99.8
317	DERRUSNIN	14736-62-0	C19H16O7	356.34		p7 2-7	14.81	6.25	4	-0.10	0.12	4	-7.3
318	SALIDROSIDE	10338-51-9	C15H22O7	314.34		p4 2-7	14.81	6.25	4	-0.22	0.12	4	-16.1
319	DIOSGENIN	512-04-9	C27H42O3	414.63	antiinflammatory, estrogen	p8 5-6	14.72	9.01	4	0.32	0.03	4	19.7
320	ESTRIOL	50-27-1, 514-68-1 [as,	C18H24O3	288.39	estrogen	p10 8-1	14.41	3.17	4	-0.12	0.03	4	-8.4
321	1-MONOPALMITIN	542-44-9	C19H38O4	330.51		p3 2-3	14.21	5.96	4	-0.10	0.12	4	-7.1
322	URSINOIC ACID	30265-59-9	C15H16O5	276.29		p6 1-3	14.19	6.68	4	-0.46	0.15	4	-37.5
323	BIOCHANIN A	491-80-5	C16H12O5	284.27	phytoestrogen	p7 7-5	14.14	1.37	4	0.10	0.06	4	6.9
324	SCOPOLAMINE HYDROBROMIDE	6533-68-2, 114-49-8 [anhydrous], 51-34-3	C17H22BrNO4	384.27	anticholinergic, treatment of motion sickness	p8 10-1	13.97	3.61	4	-0.37	0.08	4	-29.6
325	CHOLESTANE	481-21-0	C27H48	372.68		p2 6-8	13.94	4.58	4	0.19	0.10	4	12.2
326	EPIAFZELECHIN TRIMETHYL ETHER	0	C18H20O5	316.36		p2 5-3	13.83	5.29	4	0.21	0.09	4	13.4
327	DIOSMIN	520-27-4	C28H32O15	608.56	vascular protectant	p5 2-1	13.82	8.13	4	-0.65	0.08	4	-57.1
328	IRIGENIN TRIMETHYL ETHER	0	C21H22O8	402.40		p5 1-3	13.72	5.05	4	-0.48	0.11	4	-39.9
329	MUNDOSERONE	3564-85-0	C19H18O6	342.35		p2 2-2	13.71	4.46	4	0.17	0.11	4	11.4
330	COLCHICEINE	477-27-0	C21H23NO6	385.42	antimitotic	p8 8-2	13.67	3.66	4	-0.43	0.09	4	-34.9
331	SMILAGENIN	126-18-1	C27H44O3	416.65		p2 3-1	13.52	9.54	4	0.58	0.17	4	33.2
332	FORMONONETIN	485-72-3	C16H12O4	268.27	phytoestrogen	p4 7-1	13.17	6.00	4	-0.62	0.09	4	-53.6
333	3,7-EPOXYCARYOPHYLLAN-6-ONE	0	C15H24O2	236.36		p2 1-5	13.14	15.03	4	0.27	0.16	4	17.0
334	COUMARIN	91-64-5	C9H6O2	146.15	antineoplastic, antiinflammatory, antihyperglycaemic	p6 4-1	12.97	3.50	4	-0.05	0.05	4	-3.8
335	2'-METHOXYFORMONETIN	0	C17H14O5	298.30		p9 10-6	12.89	7.80	4	0.18	0.04	4	11.5
336	3,7-EPOXYCARYOPHYLLAN-6-OL	0	C15H26O2	238.37		p2 6-5	12.87	11.01	4	0.11	0.02	4	7.2
337	ASCORBIC ACID	50-81-7	C6H8O6	176.13	antiscorbutic, antiviral	p10 3-6	12.01	3.61	4	0.44	0.46	4	26.2
338	EPHEDRINE (1R,2S) HYDROCHLORIDE	50-98-6, 299-42-3 [(-)-	C10H16ClNO	201.70	bronchodilator, cardiac stimulant	p8 4-1	11.97	8.77	4	-0.06	0.14	4	-4.2
339	MONOCROTALINE	315-22-0	C16H23NO6	325.36	antineoplastic, insect sterilant	p9 9-7	11.96	2.49	4	0.14	0.08	4	9.5
340	ANHYDROBRAZILIC ACID	0	C12H10O5	234.21		p4 7-6	11.90	1.58	4	-0.01	0.07	4	-0.5
341	BIOTIN	58-85-5	C10H16N2O3S	244.31	vitamin B complex	p10 8-6	11.89	4.14	4	-0.24	0.06	4	-18.4
342	INOSITOL	87-89-8	C6H12O6	180.16	growth factor	p10 4-2	11.86	6.91	4	0.08	0.07	4	5.5
343	ROTENONIC ACID, METHYL ETHER	0	C24H26O6	410.47		p6 4-3	11.77	4.72	4	0.30	0.05	4	18.7
344	3,4-DIMETHOXYDALBERGIONE	41043-20-3	C17H16O4	284.31	induces dermatitis	p8 3-7	11.56	11.82	4	0.06	0.04	4	3.8
345	HARMALOL HYDROCHLORIDE DIHYDRATE	6028-07-5	C12H17ClN2O3	272.73	anthelmintic, narcotic agent	p8 7-8	11.47	5.47	4	-0.76	0.07	4	-69.6
346	PERSEITOL	527-06-0	C7H16O7	212.20		p2 4-2	11.32	4.16	4	-0.23	0.23	4	-17.1
347	beta-AMYRIN ACETATE	1616-93-9	C32H52O2	468.77		p5 8-4	11.26	3.57	4	0.01	0.06	4	0.5
348	QUINIDINE GLUCONATE	7054-25-3, 6591-63-5 [quinidine sulfate], 56-54-	C26H36N2O9	520.58	antiarrhythmic, antimalarial	p10 2-3	11.23	4.82	4	-0.03	0.08	4	-2.0
349	SAPPANONE A DIMETHYL ETHER	0	C18H16O5	312.33		p5 4-3	11.12	6.11	4	0.18	0.06	4	11.8
350	ANTIMYCIN A (A1 shown)	1397-94-0	C27H38N2O9	534.61	antifungal, antiviral, interferes in cytochrome oxidation	p5 4-7	10.91	5.69	4	0.12	0.15	4	7.7
351	11a-ACETOXYPROGESTERONE	2268-98-6	C23H32O4	372.51	metabolite of progesterone	p5 4-1	10.83	5.10	4	-0.22	0.08	4	-16.6
352	CATECHIN PENTAACETATE	0	C25H24O11	500.46		p9 6-6	10.66	6.03	4	-0.26	0.03	4	-19.6
353	ESCULIN MONOHYDRATE	531-75-9	C15H18O10	358.30	antiinflammatory	p8 9-7	10.62	2.38	4	-0.42	0.12	4	-34.0
354	LUPANINE PERCHLORATE	550-90-3 (base)	C15H25ClN2O5	348.83		p1 9-2	10.49	11.23	4	-0.09	0.03	4	-6.1
355	DIHYDROROTENONE	0	C23H24O6	396.44		p8 10-3	10.36	5.33	4	0.76	0.10	4	40.9
356	SHIKIMIC ACID	138-59-0	C7H10O5	174.15		p1 7-2	10.28	12.75	4	0.06	0.15	4	3.9
357	THEAFLAVIN	4670-05-7	C29H24O12	564.51	antioxidant	p4 8-5	10.19	1.90	4	0.11	0.06	4	7.6
358	SEROTONIN HYDROCHLORIDE	50-67-9	C10H13ClN2O	212.68	neurotransmitter	p3 6-1	10.18	7.38	4	-0.40	0.05	4	-31.7
359	HEMATEIN	475-25-2	C16H12O6	300.27		p1 9-3	10.13	3.46	4	0.13	0.03	4	8.6
360	EPIGALLOCATECHIN-3-MONOGALLATE	989-51-5	C22H18O11	458.38		p2 6-3	10.02	3.33	4	-0.19	0.24	4	-14.2

361	TETRAHYDROSAPPANONE A TRIMETHYL ETHER	0	C19H22O5	330.38		p8 2-5	9.90	14.12	4	-0.10	0.10	4	-6.9
362	4'-HYDROXYCHALCONE	2657-25-2	C15H12O2	224.26		P2 8-6	9.45	17.15	4	0.33	0.24	4	20.5
363	SANTONIN	481-06-1	C15H18O3	246.31		P2 10-3	9.41	6.60	4	0.18	0.08	4	11.8
364	CEDRYL ACETATE	77-54-3	C17H28O2	264.41		p2 5-7	9.38	7.19	4	0.05	0.26	4	3.6
365	PALMATINE CHLORIDE	10605-02-4	C21H22ClNO4	387.87	antibacterial, antimalarial, uterine contractant	p3 8-4	9.29	3.91	4	-0.09	0.06	4	-6.1
366	7-HYDROXYFLAVONE	6665-86-7	C15H10O3	238.25	antifungal, analgesic	p7 3-6	9.24	3.62	4	0.17	0.05	4	10.8
367	SECURININE	5610-40-2	C13H15NO2	217.27	GABAA receptor blocker, CNS stimulant	p4 3-6	9.24	3.62	4	0.05	0.05	4	3.6
368	LAGOCHILIN	23554-81-6	C20H36O5	356.51		P2 7-1	9.20	3.84	4	0.60	0.13	4	34.1
369	ROBUSTIC ACID	5307-59-5	C22H20O6	380.40		p5 9-7	9.13	7.61	4	0.28	0.10	4	17.8
370	PHENACYLAMINE HYDROCHLORIDE	5468-37-1, 613-89-	C8H10ClNO	171.63		p8 10-4	9.11	3.46	4	1.22	0.29	4	57.0
371	OROTIC ACID	65-86-1	C5H4N2O4	156.10	hepatoprotectant, uricosuric agent	p6 6-5	8.85	2.92	4	-0.39	0.14	4	-31.1
372	ANABASAMINE HYDROCHLORIDE	20410-87-1(base)	C16H20ClN3	289.81		P2 8-1	8.75	22.46	4	0.40	0.11	4	24.3
373	FARNESOL	4602-84-0	C15H26O	222.37		p5 8-6	8.75	3.76	4	0.02	0.03	4	1.0
374	ARABITOL	7643-75-6	C5H12O5	152.15		p2 5-4	8.73	8.93	4	-0.04	0.10	4	-2.6
375	CITRININ	518-75-2	C13H14O5	250.25	antibacterial	p1 3-7	8.51	0.32	2	0.13	0.11	2	8.3
376	ANTIAROL	642-71-7	C9H12O4	184.19		p4 7-5	8.48	1.70	4	0.10	0.04	4	6.9
377	ARISTOLOCHIC ACID	313-67-7	C17H11NO7	341.28	PLA2 inhibitor	p9 6-2	8.39	7.27	4	-0.03	0.24	4	-1.8
378	HYDROLYSIS PRODUCT OF BUSSEIN	0	C32H40O14	648.67		p2 4-8	8.30	8.98	4	-0.69	0.03	4	-60.9
379	DEHYDROABIETAMIDE	0	C20H29NO	299.46		p7 2-5	8.30	1.98	4	-0.01	0.04	4	-0.7
380	BRAZILIN	474-07-7	C16H14O5	286.29		p4 2-5	8.30	1.98	4	-0.12	0.04	4	-8.9
381	ONONETIN	487-49-0	C15H14O4	258.28		p9 7-2	8.27	4.90	4	-0.18	0.11	4	-12.9
382	ALLANTOIN	97-59-6	C4H6N4O3	158.12	wound healing agent	p10 1-5	8.13	3.78	4	-0.22	0.14	4	-16.1
383	2-HYDROXY-3,4-DIMETHOXYBENZOIC ACID	5653-46-3	C9H10O5	198.18	prostaglandin synthetase inhibitor	p7 4-7	7.69	4.70	4	0.16	0.07	4	10.5
384	3-DESHYDROXYSAPPANOL TRIMETHYL ETHER	0	C19H22O5	330.38		p4 4-7	7.69	4.70	4	0.05	0.07	4	3.2
385	TRETINOIN	302-79-4	C20H28O2	300.44	keratolytic, antiacne, antineoplastic	p10 2-8	7.49	5.74	4	-0.28	0.02	4	-21.5
386	SCLAREOL	515-03-7	C20H36O2	308.51	antineoplastic, apoptosis inducer	p6 1-6	7.29	10.13	4	-0.15	0.05	4	-10.7
387	DEHYDROEPIANDROSTERONE	53-43-0	C19H28O2	288.43		p4 10-8	7.05	3.42	4	-0.03	0.06	4	-1.9
388	EPIGALLOCATECHIN	970-74-1	C15H14O7	306.27		p2 5-5	7.01	3.36	4	0.15	0.06	4	9.9
389	ANABASINE HYDROCHLORIDE	13078-04-1 (anabasine)	C10H15ClN2	198.70	insecticide	p3 8-7	6.88	6.75	4	-0.08	0.11	4	-5.5
390	BERBAMINE HYDROCHLORIDE	478-61-5 (berbamine)	C37H42Cl2N2O6	681.66	antihypertensive, skeletal muscle relaxant	p8 6-8	6.86	18.93	4	-0.21	0.12	4	-15.6
391	GENISTEIN	446-72-0	C15H10O5	270.24	increases bone mineral density	p6 3-3	6.85	14.63	4	0.20	0.01	4	12.7
392	3beta-HYDROXYISOALLOSPIROST-9(11)-ENE	0	C27H42O3	414.63		p6 7-7	6.79	10.10	4	-0.17	0.06	4	-12.6
393	RETINOL	68-26-8	C20H30O	286.46	vitamin A	p10 1-6	6.73	3.26	4	0.09	0.07	4	6.3
394	AZELAIC ACID	123-99-9	C9H16O4	188.23	antiacne, antiproliferative agent	p10 3-4	6.72	8.45	4	0.03	0.05	4	1.8
395	HECOGENIN ACETATE	915-35-5	C29H44O5	472.67		p1 9-5	6.66	5.55	4	-0.05	0.08	4	-3.7
396	FUCOSTANOL	83-45-4	C29H52O	416.74		p1 7-5	6.66	5.55	4	-0.69	0.15	4	-61.6
397	GEDUNIN	2753-30-2	C28H34O7	482.58	antifeedant; heat shock inducer	p8 7-6	6.58	8.52	4	0.02	0.03	4	1.2
398	EPICATECHIN MONOGALLATE	1257-08-5	C22H18O10	442.38		p2 3-3	6.24	5.97	4	0.38	0.08	4	23.1
399	LINAMARIN	554-35-8	C10H17NO6	247.25		p1 6-6	6.23	7.26	4	0.59	0.08	4	33.5
400	ADONITOL	488-81-3	C5H12O5	152.15		P2 7-4	6.19	1.71	4	0.26	0.11	4	16.4
401	2',4'-DIHYDROXYCHALCONE	1776-30-3	C15H12O3	240.26	anthelmintic, antiulcer	p7 2-6	6.05	1.97	4	0.09	0.10	4	6.1
402	KOPARIN	65048-75-1	C16H12O6	300.27		p4 2-6	6.05	1.97	4	-0.02	0.10	4	-1.6
403	HOMOPTEROCARPIN	606-91-7	C17H16O4	284.31		p3 3-5	5.57	3.90	4	-0.01	0.09	4	-0.7
404	ANISODAMINE HYDROBROMIDE	17659-49-3	C17H23NO4	305.38	anticholinergic, antispasmodic	p5 1-2	5.51	11.32	4	-0.41	0.17	4	-32.6
405	YOHIMBINE HYDROCHLORIDE	65-19-0	C21H27ClN2O3	390.91	alpha adrenergic blocker, mydriatic, antidepressant	p10 6-4	5.49	13.70	4	-0.45	0.07	4	-36.5
406	LUPANYL ACID HYDROCHLORIDE	0	C14H25ClN2O2	288.82		p5 7-5	5.40	7.81	4	0.09	0.15	4	6.0
407	CYSTAMINE DIHYDROCHLORIDE	56-17-7	C4H14Cl2N2S2	225.20	hepatoprotectant, radioprotectant	p6 6-6	5.08	1.87	4	-0.15	0.05	4	-11.2
408	GARLICIN	2179-57-9	C6H10S2	146.27	antineoplastic, antibacterial, apoptosis inducer, insecticide	p5 1-6	4.93	13.44	4	-0.39	0.07	4	-30.8
409	GLUCITOL-4-GUCOPYANOSIDE	0	C12H24O11	344.32		p9 4-5	4.89	7.96	4	-0.19	0.06	4	-14.4
410	AVOCATIN B	0	C34H66O6	570.90		p7 10-5	4.87	5.74	4	0.36	0.07	4	21.9

411	DIHYDROFISSINOLIDE	0	C29H38O8	514.62		p4 9-2	4.50	2.93	4	0.08	0.05	4	5.3
412	OMEGA-3-ACID ESTERS (EPA shown)	86227-47-6	C22H34O2	330.52	hypolipidemic	p6 1-7	4.36	14.00	4	-0.23	0.06	4	-17.4
413	DIHYDROMUNDULETONE	0	C25H28O6	424.50		p2 6-7	4.35	5.15	4	-0.10	0.03	4	-7.4
414	BEKANAMYCIN SULFATE	4696-76-8 (base)	C18H39N5O14S	581.60	antibacterial	p10 3-5	4.16	9.06	4	0.13	0.07	4	8.7
415	PICEID	27208-80-6	C20H22O8	390.39	antioxidant	p5 2-7	4.10	0.69	4	-0.36	0.13	4	-28.7
416	TOBRAMYCIN	32986-56-4	C18H37N5O9	467.52	antibacterial, inhibits protein synthesis	p10 9-3	4.08	4.67	4	-0.49	0.19	4	-40.8
417	GRAYANOTOXIN I	4720-09-6	C22H36O7	412.53		p1 1-3	3.95	7.58	4	-0.03	0.26	3	-2.4
418	CAFFEINE	58-08-2, 5743-12-4	C8H10N4O2	194.19	CNS stimulant	p2 1-1	3.94	4.95	4	0.27	0.08	4	16.9
419	AURAPTENE	495-02-3	C19H22O3	298.39	antineoplastic, apoptosis inducer	p5 9-1	3.93	6.03	4	0.02	0.12	4	1.2
420	ACTEIN	18642-44-9	C37H56O11	676.85	antihypertensive	p8 9-1	3.90	15.38	4	-0.41	0.09	4	-33.3
421	QUERCITRIN	522-12-3	C21H20O11	448.39	antihemmorrhagic	p2 2-1	3.89	5.08	4	-0.65	0.46	4	-57.2
422	CINCHONIDINE	485-71-2	C19H22N2O	294.40	antimalarial	p8 4-7	3.76	12.00	4	-0.20	0.06	4	-15.2
423	ATROPINE SULFATE	51-55-8, 5908-99-6 [atropine sulfate], 55-48-1	C17H25NO7S	387.46	anticholinergic, mydriatic	P2 9-1	3.70	7.88	4	0.26	0.03	4	16.3
424	FOLIC ACID	59-30-3	C19H19N7O6	441.41	hematopoietic vitamin	p6 8-7	3.63	3.37	4	0.02	0.05	4	1.3
425	SYRINGIC ACID	530-57-4	C9H10O5	198.18		p3 8-1	3.63	5.94	4	-0.26	0.07	4	-20.0
426	THYMOQUINONE	0	C10H12O2	164.21		p8 1-4	3.13	18.98	4	-0.08	0.07	4	-5.8
427	EUGENOL	97-53-0	C10H12O2	164.21	analgesic (topical), antiseptic, antifungal	p10 10-1	3.09	5.56	4	-0.36	0.04	4	-28.5
428	SAPPANONE A TRIMETHYL ETHER	0	C19H18O5	326.35		p6 9-5	3.07	5.23	4	0.23	0.04	4	14.7
429	HYMECHROME	90-33-5	C10H8O3	176.17	choloretic, spasmolytic, sunscreen	p7 3-1	2.86	6.06	4	-0.28	0.01	4	-21.2
430	CINNAMIC ACID	621-82-9	C9H8O2	148.16	fragrance & flavoring agent	p4 3-1	2.86	6.06	4	-0.39	0.01	4	-31.0
431	ACONITINE	302-27-2	C34H47NO11	645.75	anesthetic (gastric), antipyretic, and cardiotoxin	p10 7-2	2.84	6.92	4	-0.16	0.07	4	-11.7
432	GALANTAMINE	357-70-0, 1953-04-4 [hydrobromide]	C17H21NO3	287.36	anticholinesterase, analgesic, antiAlzheimer	p6 6-7	2.66	4.46	4	-0.34	0.08	4	-26.2
433	PHENYLALANIINE (L) HYDROCHLORIDE	63-91-2(base)	C9H12ClNO2	201.65	amino acid	p9 10-7	2.59	10.09	4	-0.12	0.08	4	-8.5
434	4-O-METHYLPHLORACETOPHENONE	7507-89-3	C9H10O4	182.18	antifungal	p4 9-7	2.57	9.05	4	0.16	0.12	4	10.4
435	ERGONOVINE MALEATE	129-51-1, 60-79-7	C23H27N3O6	441.49	oxytocic, 5HT antagonist	p6 4-7	2.56	2.61	4	0.23	0.07	4	14.5
436	ISOROTENONE	0	C23H22O6	394.43		p1 2-7	2.53	6.12	4	0.71	0.16	4	38.9
437	GLUTAMINE (D)	6899-04-3	C5H10N2O3	146.15		p6 1-2	2.46	13.58	4	-0.16	0.06	4	-11.8
438	PURPUROGALLIN-4-CARBOXYLIC ACID	0	C12H8O7	264.19	antioxidant	p2 3-5	2.20	8.17	4	0.11	0.17	4	7.3
439	URACIL	66-22-8	C4H4N2O2	112.09	antineoplastic	p7 2-3	2.08	6.08	4	-0.28	0.05	4	-21.4
440	PEUCENIN	578-72-3	C15H16O4	260.29		p4 2-3	2.08	6.08	4	-0.39	0.05	4	-31.3
441	NARINGIN	10236-47-2	C27H32O14	580.55	antihaemorrhagic, antiinflammatory	p7 8-4	2.06	3.69	4	0.16	0.06	4	10.4
442	ISOBERGAPTENE	482-48-4	C12H8O4	216.20		p2 6-6	2.00	6.85	4	0.17	0.08	4	11.0
443	CIANIDANOL	154-23-4	C15H14O6	290.28	procollagen production inhibitoe, hepatoprotectant	p6 6-4	2.00	2.91	4	-0.20	0.10	4	-14.9
444	BACCATIN III	27548-93-2	C31H38O11	586.64		P2 8-5	1.76	6.79	4	0.08	0.03	4	5.2
445	MENTHYL BENZOATE	0	C17H24O2	260.38		p7 3-4	1.74	4.94	4	0.24	0.06	4	15.1
446	XANTHYLETIN	553-19-5	C14H12O3	228.25		p4 3-4	1.74	4.94	4	0.12	0.06	4	8.1
447	APOTOXICAROL	0	C18H14O7	342.31		p7 9-4	1.64	2.64	4	0.16	0.07	4	10.7
448	CAMPHOR (1R)	464-49-3; 76-22-2	C10H16O	152.24	analgesic, antiinfective, antipruritic	p7 4-3	1.63	3.26	4	-0.22	0.04	4	-16.2
449	OBLIQUIN	0	C14H12O4	244.25		p4 4-3	1.63	3.26	4	-0.33	0.04	4	-25.7
450	RHODOCLADONIC ACID	26984-15-6	C15H10O8	318.24		p1 8-2	1.58	7.53	4	-0.03	0.09	4	-2.1
451	ROCCELLIC ACID	22139-54-4	C17H32O4	300.44		p1 10-2	1.58	7.53	4	-0.06	0.05	4	-4.1
452	HYGROMYCIN B	31282-04-9	C20H37N3O13	527.53	antibacterial, anthelmintic; LD5 ip (rat) 63mg/Kg	p6 4-2	1.53	2.76	4	0.15	0.02	4	9.9
453	GLYCYRRHIZIC ACID, AMMONIUM SALT	1405-86-3	C41H69N3O16	860.02	11beta-hydroxysteroid dehydrogenase inhibitor, antiinflammatory, expectorant, antihaemorrhagic, anti-HIV	p8 8-7	1.41	3.60	4	-0.52	0.13	4	-43.6
454	7,4'-DIMETHOXYISOFLAVONE	0	C17H14O4	282.30		p8 5-3	1.31	16.35	4	0.04	0.08	4	3.0
455	6,3'-DIMETHOXYFLAVONE	79786-40-6	C17H14O4	282.30		p9 8-6	1.27	13.51	4	-0.08	0.11	4	-5.6
456	alpha-TOXICAROL (dl)	82-09-7	C23H22O7	410.43		p7 2-4	0.92	7.28	4	0.01	0.16	4	0.6
457	2-METHYL GRAMINE	0	C12H16N2	188.27		p4 2-4	0.92	7.28	4	-0.11	0.16	4	-7.5
458	PIPERIC ACID	5285-18-7	C12H10O4	218.21		p3 3-6	0.79	4.72	4	-0.02	0.07	4	-1.7

459	INDOLE-3-CARBINOL	700-06-1	C9H9NO	147.18	antineoplastic; inhibitor of Amyloid-beta deposition	p5 1-5	0.77	4.37	4	-0.40	0.12	4	-31.7
460	OXONITINE	0	C33H43NO12	645.71		p4 8-3	0.75	4.78	4	0.05	0.05	4	3.3
461	12a-HYDROXY-9-DEMETHYLMUNDUSERONE-8-CARBOXYLIC ACID	0	C19H16O9	388.33		p4 8-4	0.54	6.13	4	0.06	0.02	4	4.1
462	SINAPIC ACID	530-59-6	C11H12O5	224.22		p7 5-7	0.45	3.04	4	0.25	0.13	4	15.8
463	XANTHONE	90-47-1	C13H8O2	196.21		p4 5-7	0.45	3.04	4	0.13	0.13	4	8.9
464	PERSEITOL HEPTAACETATE	19147-10-5	C21H30O14	506.46		p9 5-5	0.17	3.78	4	-0.14	0.04	4	-10.1
465	CHOLECALCIFEROL	67-97-0	C27H44O	384.65	vitamin D3	p10 6-5	0.08	17.94	4	-0.21	0.08	4	-15.8
466	AVOCADENE ACETATE	24607-09-8	C19H36O4	328.50	antifungal, plant growth inhibitor	p9 1-4	-0.39	8.75	4	-0.47	0.14	4	-38.4
467	STICTIC ACID	549-06-4	C19H14O9	386.32		P2 7-8	-0.45	10.21	4	0.08	0.15	4	5.5
468	BICUCULLINE(-) METHIODIDE	55950-07-7	C21H20INO6	509.30	GABAa antagonist	p7 9-1	-0.49	3.50	4	-0.07	0.12	4	-4.9
469	EPICATECHIN	490-46-0	C15H14O6	290.28	antioxidant	p9 10-1	-0.62	3.01	4	-0.09	0.04	4	-6.3
470	ALOIN	5133-19-7	C21H22O10	434.40	cathartic, laxative	p6 6-1	-0.73	14.69	4	-0.63	0.12	4	-54.3
471	CLOVANEDIOL DIACETATE	0	C19H30O4	322.45		P2 10-6	-0.83	15.10	4	0.55	0.08	4	31.9
472	CHAULMOOGRIC ACID, ETHYL ESTER	623-32-5	C20H36O2	308.51	antilepretic	p6 7-6	-0.95	4.76	4	-0.15	0.02	4	-10.8
473	DIPTERYXIN	53948-01-9	C17H14O6	314.30		p1 6-3	-1.01	12.29	4	-0.31	0.14	4	-24.3
474	EPIGALLOCATECHIN 3,5-DIGALLATE	0	C29H22O15	610.49		p9 8-3	-1.19	4.93	4	-0.23	0.04	4	-17.5
475	AJMALINE	4360-17-7	C20H26N2O2	326.44	antiarrhythmic (Class Ia): inhibits glucose uptake by mitochondria, & PAF blocker	p3 10-6	-1.24	6.91	4	0.10	0.08	4	6.9
476	SALSOLINE	89-31-6	C11H15NO2	193.25	antihypertensive, antihistamine	p9 8-5	-1.38	13.01	4	-0.01	0.09	4	-0.9
477	HYMECROMONE METHYL ETHER	2555-28-4	C11H10O3	190.20		P2 9-2	-1.44	21.29	4	0.26	0.05	4	16.6
478	OSAJIN	482-53-1	C25H24O5	404.47		p2 2-3	-1.77	15.55	4	-1.06	0.54	4	-108.2
479	PICROTIN	21416-53-5	C15H18O7	310.31	GABAa receptor antagonist	p5 3-5	-1.94	5.37	4	-0.20	0.17	4	-14.8
480	EPIAFZELECHIN (2R,3R)(-)	24808-04-6	C15H14O5	274.28		p3 3-7	-2.10	11.70	4	-0.14	0.18	4	-10.2
481	MANDELIC ACID, METHYL ESTER	0	C9H10O3	166.18		p2 1-2	-2.26	2.60	4	0.31	0.16	4	19.1
482	CONESSINE	5913-82-6, 546-06-5 [conessine]	C24H40N2	356.60	antiamebic, antibacterial, antineoplastic, anesthetic (local)	p9 8-4	-2.33	11.69	4	-0.14	0.07	4	-10.4
483	HIERACIN	1621-84-7	C15H10O7	302.24		p1 3-6	-2.75	15.90	2	0.54	0.22	2	31.0
484	EUPHOL	514-47-6	C30H50O	426.73		p9 8-7	-2.84	9.90	4	-0.29	0.04	4	-22.5
485	MENADIONE	58-27-5	C11H8O2	172.19	prothrombogenic agent	p10 4-6	-2.86	6.60	4	0.49	0.07	4	29.0
486	AGMATINE SULFATE	2482-00-0	C5H16N4O4S	228.27	NMDA blocker, alpha-2 adrenergic agonist; NO synthase inhibitor	p5 10-7	-2.93	14.78	4	0.21	0.17	4	13.5
487	MUNDULONE ACETATE	0	C28H28O7	476.53		p6 7-3	-3.19	6.82	4	0.37	0.04	4	22.6
488	CANAVANINE	543-38-4	C5H12N4O3	176.18	NO synthase inhibitor	p5 5-6	-3.40	5.55	4	0.29	0.05	4	18.4
489	CARNITINE (dl) HYDROCHLORIDE	461-06-3	C7H16ClNO3	197.66	antihyperlipoproteinemic, gastric/ pancreatic secretion stimulant	p8 6-1	-3.41	16.17	4	-0.79	0.04	4	-73.4
490	RIBOFLAVIN 5-PHOSPHATE SODIUM	130-40-5	C17H20N4NaO9P	478.33	vitamin, enzyme cofactor	p10 8-7	-3.47	5.89	4	-0.32	0.08	4	-25.0
491	MANGIFERIN	4773-96-0	C19H18O11	422.35	MAO inhibitor, immunostimulant	p1 3-2	-3.49	11.57	4	0.18	0.20	4	11.5
492	N-METHYLISOLEUCINE	5125-98-8	C7H15NO2	145.20		p9 9-1	-3.51	17.39	4	-0.07	0.05	4	-5.2
493	ACONITIC ACID	585-84-2	C6H6O6	174.11		P2 7-7	-3.59	5.97	4	-0.01	0.10	4	-0.7
494	4-METHYLESCULETIN	0	C10H8O4	192.17		p8 2-7	-3.64	20.21	4	0.01	0.13	4	0.7
495	LANOSTEROL ACETATE	0	C32H52O2	468.77		p3 1-2	-3.78	4.86	4	-0.04	0.16	4	-2.8
496	DIFFRACTAIC ACID	436-32-8	C20H22O7	374.39		p1 5-6	-3.80	8.68	4	0.91	0.21	4	46.7
497	SITOSTERYL ACETATE	0	C31H52O2	456.76		p4 7-2	-3.86	7.26	4	-0.17	0.12	4	-12.4
498	PANGAMIC ACID SODIUM	20858-86-0	C10H18NNaO8	303.25		p8 8-4	-4.04	4.03	4	-0.69	0.11	4	-61.0
499	METHYL 7-DESHYDROXYPYROGALLIN-4-CARBOXYLATE	77-41-8	C13H10O6	262.22		p3 9-3	-4.08	8.73	4	0.16	0.16	4	10.5
500	AMPHOTERICIN B	1397-89-3	C47H73NO17	924.10	antifungal	p7 9-3	-4.47	6.51	4	0.11	0.06	4	7.1
501	VERBENALIN	548-37-8	C17H24O10	388.37	cell growth inhibitor, coagulant	p8 9-3	-4.48	7.07	4	2.45	1.77	4	81.7
502	AGARIC ACID	666-99-9	C22H40O7	416.56	antiperspirant	p3 9-5	-4.50	6.51	4	0.30	0.07	4	18.9
503	CEDROL	77-53-2	C15H26O	222.37	acaricide	p7 6-4	-4.53	8.49	4	-0.11	0.07	4	-7.7
504	SENNOSIDE A	81-27-6	C42H38O20	862.76	cathartic	p5 6-2	-4.71	12.19	4	-0.08	0.11	4	-6.1
505	PHYSOSTIGMINE SALICYLATE	57-64-7, 57-47-6	C22H27N3O5	413.48	cholinergic, anticholinesterase, miotic	p10 9-2	-4.89	6.86	4	-0.12	0.04	4	-8.6
506	PODOTOTARIN	0	C40H58O2	570.91		p6 9-2	-4.91	3.66	4	0.04	0.06	4	2.5

507	CHAULMOOGRIC ACID	502-30-7	C18H32O2	280.45	antibacterial (mycobacteria), antileprotic	p1 5-1	-4.94	10.64	4	-0.58	0.08	4	-49.1
508	GALANGIN 3-O-METHYL ETHER	6665-74-3	C16H12O5	284.27		p5 2-8	-5.04	2.62	4	-0.54	0.10	4	-45.3
509	2',4-DIHYDROXY-3,4',6'-TRIMETHOXYCHALCONE	112572-59-5	C18H18O6	330.34		p7 4-6	-5.12	7.47	4	0.09	0.03	4	5.9
510	PISCIDIC ACID	35388-57-9	C11H12O7	256.21		p4 4-6	-5.12	7.47	4	-0.03	0.03	4	-1.7
511	CORALYNE CHLORIDE	38989-38-7	C22H22ClNO4	399.88	cytostatic, intercalating agent	p9 2-1	-5.22	5.70	4	-0.31	0.06	4	-23.6
512	ANETHOLE	4180-23-8	C10H12O	148.21	expectorant, gastric stimulant, insecticide	p10 10-6	-5.30	5.83	4	-0.08	0.06	4	-5.8
513	CAPSANTHIN	465-42-9	C40H56O3	584.89	antineoplastic	p3 8-3	-5.31	4.63	4	-0.08	0.05	4	-5.5
514	EMBELIN	0	C17H26O4	294.39	anthelmintic, oral contraceptive	p6 4-5	-5.38	6.03	4	0.13	0.07	4	8.8
515	GLUCOSAMINIC ACID	3646-68-2	C6H13NO6	195.17		p7 4-5	-5.48	1.58	4	0.22	0.03	4	13.9
516	FUMARPROTOCETRARIC ACID	489-50-9	C22H16O12	472.37		p4 4-5	-5.48	1.58	4	0.10	0.03	4	6.9
517	SPHONDIN	483-66-9	C12H8O4	216.20		p7 9-5	-5.49	8.01	4	0.32	0.09	4	20.1
518	CELLOBIOSE (D[+])	528-50-7	C12H22O11	342.30		p1 4-5	-5.56	11.11	4	0.91	0.15	4	46.9
519	PHLORETIN	60-82-2	C15H14O5	274.28		p1 9-1	-5.73	13.60	4	-0.15	0.06	4	-10.8
520	PSEUDO-ANISATIN	31090-37-6	C15H22O6	298.34	GABA antagonist	p9 9-6	-5.73	8.27	4	0.14	0.09	4	9.2
521	2,6-DIMETHOXYQUINONE	35069-70-6	C8H8O4	168.15	antibacterial, induces dermatitis, mutagen	p9 7-3	-5.86	5.05	4	-0.06	0.05	4	-4.3
522	KHAYANTHONE	25279-68-9	C32H42O9	570.69		p3 9-4	-5.94	1.06	4	-0.01	0.07	4	-0.3
523	SPECTINOMYCIN HYDROCHLORIDE	22189-32-8, 21736-83-4 [anhydrous], 1695-77-8 [spectinomycin]	C14H26Cl2N2O7	405.28	antibacterial	p10 6-3	-6.16	18.84	4	-0.51	0.13	4	-42.8
524	3-PINANONE OXIME	0	C10H17NO	167.25		p6 7-4	-6.18	3.64	4	0.02	0.11	4	1.5
525	DESACETYLCOLFORSIN	64657-20-1	C20H32O6	368.47		p9 9-4	-6.53	4.67	4	0.22	0.03	4	14.3
526	d-LIMONENE	138-86-7	C10H16	136.24	skin irritant, sensitizer	p6 6-2	-6.54	13.29	4	-0.21	0.12	4	-15.7
527	2',3-DIHYDROXY-4,4',6'-TRIMETHOXYCHALCONE	38186-71-9	C18H18O6	330.34		p1 2-1	-6.64	4.60	4	-0.29	0.13	4	-22.2
528	NIACIN	59-67-6	C6H5NO2	123.11	antihyperlipidemic, vitamin (enzyme cofactor)	p6 3-7	-6.70	6.44	4	0.08	0.09	4	5.3
529	ARECOLINE HYDROBROMIDE	300-08-3, 63-75-2	C8H14BrNO2	236.11	anthelmintic (Cestodes), hypotensive, cathartic	p7 1-1	-6.72	12.00	4	-0.47	0.05	4	-38.7
530	PRENYLETIN	15870-91-4	C14H14O4	246.27		p4 1-1	-6.72	12.00	4	-0.59	0.05	4	-50.0
531	STRYCHNINE METHIODIDE	0	C22H25IN2O2	476.36	neuromuscular blocker	P2 7-2	-6.90	10.25	4	0.58	0.18	4	33.3
532	CARYOPHYLLENE [t(-)]	87-44-5	C14H22	190.33		p1 8-3	-6.90	16.44	4	0.32	0.16	4	20.0
533	ISOPOMIFERIN	0	C25H24O6	420.47		p1 10-3	-6.90	16.44	4	-0.07	0.15	4	-5.3
534	alpha-HYDROXYDEOXYCHOLIC ACID	83-49-8	C24H40O4	392.58		p1 5-2	-6.93	15.75	4	-0.52	0.10	4	-43.0
535	1R,2S-PHENYLPROPYLAMINE	14838-15-4	C9H13NO	151.21	decongestant	p6 8-5	-7.00	2.40	4	-0.16	0.08	4	-12.0
536	ABSCISIC ACID (cis,trans; +/-)	14375-45-2	C15H20O4	264.32	abscission-accelerant; kinetin neucleotide synthesis inhibitor	p1 6-5	-7.09	30.70	4	0.65	0.45	3	36.3
537	PALMIDROL	544-31-0	C18H37NO2	299.50	antiinflammatory	p6 5-6	-7.35	5.77	4	0.05	0.07	4	3.5
538	ISOFORMONONETIN	486-63-5	C16H12O4	268.27		p2 4-1	-7.36	15.21	4	-0.28	0.22	4	-21.7
539	EVOXINE	522-11-2	C18H21NO6	347.37		p1 3-1	-7.37	7.96	4	0.11	0.26	3	7.0
540	SPERMINE	71-44-3	C10H26N4	202.35	immune modulator	p1 5-3	-7.42	7.35	4	-0.43	0.20	4	-35.1
541	BILIRUBIN	635-65-4	C33H36N4O6	584.68		p1 8-4	-7.55	9.31	4	-0.01	0.11	4	-0.9
542	MUUROLLADIE-3-ONE	0	C15H22O	218.34		p1 10-4	-7.55	9.31	4	-0.02	0.07	4	-1.7
543	MANGOSTIN TRIMETHYL ETHER	0	C27H32O6	452.55		p3 5-6	-8.08	6.81	4	0.00	0.03	4	-0.1
544	DIHYDROJASMONIC ACID, METHYL ESTER	24851-98-7	C13H22O3	226.32	plant growth regulator	p8 3-2	-8.77	11.62	4	-0.06	0.07	4	-4.0
545	ENOXOLONE	471-53-4	C30H46O4	470.70	antitussive, antiinflammatory, antibacterial	p6 3-6	-8.81	2.02	4	-0.07	0.05	4	-5.3
546	CHRYSAROBIN	491-58-7	C15H12O3	240.26		p7 10-6	-8.96	6.12	4	0.48	0.07	4	28.2
547	GENKWANIN	437-64-9	C16H12O5	284.27		p8 6-2	-9.13	28.74	4	-0.83	0.06	4	-77.7
548	ADENINE	73-24-5	C5H5N5	135.13	Vitamin B4	p10 2-5	-9.27	4.53	4	-0.08	0.12	4	-5.9
549	EPITHEAFLAVIC ACID	0	C21H16O10	428.36		p1 1-8	-9.36	20.34	4	0.10	0.04	4	6.9
550	TOTAROL	511-15-9	C20H30O	286.46		p2 4-6	-9.45	5.71	4	-0.36	0.10	4	-28.4
551	3,4',5,6,7-PENTAMETHOXYFLAVONE	4472-73-5	C20H20O7	372.38		p3 8-6	-9.57	2.71	4	0.24	0.04	4	15.6
552	NIACINAMIDE	98-92-0	C6H6N2O	122.13	Vitamin B3; enzyme cofactor; anti-pellagra	p6 10-7	-9.59	6.64	4	-0.05	0.05	4	-3.6
553	LEVODOPA	59-92-7	C9H11NO4	197.19	antiparkinsonian	p7 6-1	-9.67	12.17	4	-0.21	0.16	4	-15.4
554	PAEONOL	552-41-0	C9H10O3	166.18	antibacterial	p2 3-7	-10.37	19.61	4	0.01	0.12	4	0.8
555	CYCLOSERINE (D)	68-41-7	C3H6N2O2	102.09	antibacterial (tuberculostatic)	p10 1-1	-10.45	9.82	4	-0.38	0.13	4	-30.0
556	CINCHONINE	118-10-5	C19H22N2O	294.40	antimalarial	p8 5-4	-10.47	18.05	4	0.01	0.12	4	0.4

557	VERATRIC ACID	93-07-2	C9H10O4	182.18		p9 4-4	-10.65	2.87	4	-0.09	0.02	4	-6.7
558	ERGOSTEROL ACETATE	2418-45-3	C32H50O2	466.75		p5 8-1	-10.68	11.05	4	0.06	0.12	4	3.7
559	DIFUCOL HEXAMETHYL ETHER	14262-07-8	C18H22O6	334.37		p3 4-7	-10.90	5.62	4	0.08	0.09	4	5.1
560	CORTISONE	53-06-5	C21H28O5	360.45	antiinflammatory, glucocorticoid	p6 2-6	-10.93	6.03	4	-0.17	0.07	4	-12.8
561	DEOXYSAPPANONE B 7,3'-DIMETHYL ETHER	0	C18H18O5	314.34		p5 9-2	-11.00	6.96	4	0.03	0.04	4	2.2
562	8-HYDROXYCARAPINIC ACID	0	C26H30O8	470.52		p1 6-7	-11.03	10.14	4	1.43	0.22	4	63.0
563	2',2'-BISEPIGALLOCATECHIN DIGALLATE	0	C44H34O22	914.75		p6 1-1	-11.23	5.23	4	-0.51	0.10	4	-42.3
564	7-AMINOCEPHALOSPORANIC ACID	957-68-6	C10H12N2O5S	272.28		p1 3-3	-11.44	6.56	4	0.19	0.26	4	12.1
565	KHELLIN	82-02-0	C14H12O5	260.25	vasodilator (coronary), photosensitizer	p6 4-6	-11.76	6.37	4	0.22	0.07	4	14.1
566	POLYMYXIN B SULFATE	1405-20-5, 1404-26-8	C56H100N16O17S	1301.58	antibacterial	p10 1-3	-11.86	13.49	4	-0.20	0.12	4	-15.1
567	DIHYDROFOLIC ACID	4033-27-6	C19H21N7O6	443.42	antidote to methotrexate toxicity	p7 1-3	-11.92	5.99	4	-0.36	0.07	4	-28.7
568	XYLOCARPUS A	0	C31H38O11	586.64		p4 1-3	-11.92	5.99	4	-0.48	0.07	4	-39.2
569	PIPERINE	94-62-2	C17H19NO3	285.35	analeptic, antibacterial	p10 8-5	-12.09	6.44	4	0.03	0.07	4	2.1
570	BRAZILEIN	600-76-0	C16H12O5	284.27		p6 8-3	-12.20	6.49	4	0.03	0.04	4	2.2
571	SPIRAMYCIN	8025-81-8	C43H74N2O14	843.07	antibacterial	p10 10-5	-12.26	13.32	4	-0.17	0.07	4	-12.2
572	MEROGEDUNIN	0	C21H28O4	344.45		p1 7-4	-12.28	11.90	4	-0.50	0.05	3	-41.1
573	ICARIIN	489-32-7	C33H40O15	676.68	hepatoprotective	p4 10-1	-12.33	2.62	4	-0.22	0.06	4	-16.4
574	LEVOTHYROXINE	51-48-9	C15H11I4NO4	776.88	antihypercholesterimic, thyromimetic	p10 8-2	-12.41	11.60	4	-0.03	0.01	4	-2.2
575	TOTAROL-19-CARBOXYLIC ACID, METHYL ESTER	0	C21H30O3	330.47		p8 4-6	-12.62	27.41	4	0.37	0.12	4	22.4
576	PURPUROGALLIN	569-77-7	C11H8O5	220.18	xanthine oxidase inhibitor, antioxidant	p3 3-2	-12.64	8.05	4	-0.01	0.10	4	-0.7
577	18alpha-GLYCYRRHETINIC ACID	0	C30H46O4	470.70	antiinflammatory	p5 4-6	-12.69	1.33	4	-0.01	0.05	4	-0.5
578	ISOPEONOL	493-33-4	C9H10O3	166.18		p7 3-7	-12.83	6.96	4	0.03	0.07	4	2.3
579	N-METHYLBENZYLAMINE HYDROCHLORIDE	13426-94-3, 103-67-3(base)	C8H12ClN	157.64		p4 3-7	-12.83	6.96	4	-0.08	0.07	4	-5.7
580	PHYTOL	0	C19H38O	282.51		p3 6-2	-12.94	12.06	4	-0.42	0.12	4	-33.6
581	BAICALIN	21967-41-9	C21H18O11	446.37	diuretic	p1 8-8	-13.01	3.92	4	-0.23	0.21	4	-17.1
582	SMILAGENIN ACETATE	0	C29H46O4	458.69		p1 1-4	-13.03	6.86	4	-0.11	0.13	4	-7.9
583	IRIDIN	491-74-7	C24H26O13	522.47		p5 3-2	-13.32	6.11	4	-0.14	0.08	4	-10.0
584	ADENOSINE	58-61-7	C10H13N5O4	267.25	antiarrhythmic, cardiac depressant	p3 10-5	-13.39	5.77	4	0.02	0.09	4	1.5
585	IRIGENOL	4935-93-7	C15H10O8	318.24		p5 7-3	-13.42	14.28	4	0.01	0.13	4	0.7
586	CHRYSANTHEMIC ACID	10453-89-1	C10H16O2	168.24	esters as insecticide	p8 7-5	-13.45	15.60	4	-0.32	0.12	4	-24.6
587	DUARTIN, DIMETHYL ETHER	0	C20H24O6	360.41		p5 5-4	-13.50	4.27	4	-0.22	0.07	4	-16.4
588	alpha-TOCHOPHERYL ACETATE	58-95-7	C31H52O3	472.76	vitamin E	p7 6-2	-13.55	13.85	4	-0.21	0.17	4	-15.8
589	NOVOBIOCIN SODIUM	1476-53-5, 303-81-1	C31H35N2NaO11	634.62	antibacterial	p1 3-8	-13.64	14.42	4	0.10	0.12	4	6.5
590	MENAQUINONE-4	0	C21H24O2	308.42	antioxidant, alkaline phosphatase enhancer	p7 8-5	-13.76	12.84	4	0.06	0.13	4	4.1
591	5alpha-ANDROSTAN-3,17-DIONE	0	C19H28O2	288.43	androgen	p5 5-8	-13.88	11.20	4	0.00	0.04	4	-0.1
592	CHOLESTERYL BENZOATE	604-32-0	C34H50O2	490.78		p6 4-4	-14.05	6.04	4	0.08	0.06	4	5.3
593	CHLORTETRACYCLINE HYDROCHLORIDE	64-72-2	C22H24Cl2N2O8	515.35	antibacterial, antiamebic, Ca chelator, hepatotoxic; inhibits protein synthesis	p7 10-3	-14.12	2.48	4	-0.03	0.05	4	-2.0
594	4-ACETOXYPHENOL	3233-32-7	C8H8O3	152.15	antioxidant	p3 7-1	-14.14	5.77	4	0.21	0.04	4	13.4
595	MUCIC ACID	526-99-8	C6H10O8	210.14		p8 4-3	-14.31	11.79	4	-0.19	0.08	4	-13.8
596	KYNURENINE	0	C10H12N2O3	208.22		P2 10-5	-14.37	9.37	4	0.48	0.24	4	28.1
597	COTARNINE CHLORIDE	10018-19-6, 82-54-2	C12H14ClNO3	255.70	vasoconstrictor	p4 10-3	-14.54	8.04	4	-0.20	0.08	4	-15.2
598	PRIMULETIN	491-78-1	C15H10O3	238.25		p4 8-2	-14.55	11.94	4	0.05	0.04	4	3.1
599	SPARTEINE SULFATE	6160-12-9, 299-39-8	C15H28N2O4S	332.47	oxytocic	p7 7-1	-14.55	7.38	4	0.04	0.16	4	3.0
600	ELLAGIC ACID	476-66-4	C14H6O8	302.20	hemostatic, antineoplastic, antimutagenic	p7 8-1	-14.62	5.34	4	-0.05	0.06	4	-3.6
601	SOLIDAGENONE	23534-56-7	C20H28O3	316.44		p2 1-7	-14.64	11.96	4	0.73	0.26	4	39.6
602	alpha-TOCHOPHEROL	59-02-9	C29H50O2	430.72	vitamin E, antioxidant	P2 10-4	-14.67	19.84	4	0.21	0.27	4	13.8
603	N-METHYLANTHRANILIC ACID	119-68-6	C8H9NO2	151.17		P2 8-4	-14.80	16.62	4	0.20	0.04	4	13.0
604	GALANGIN	548-83-4	C15H10O5	270.24	CYP1A1 inhibitor	P2 10-1	-14.90	21.99	4	0.39	0.07	4	23.6
605	IRETOL	0	C7H8O4	156.14		p6 1-4	-15.02	9.00	4	-0.32	0.07	4	-24.7
606	IRIGENIN	548-76-5	C18H16O8	360.32		p5 2-2	-15.03	12.03	4	-0.43	0.14	4	-34.9
607	QUININE SULFATE	6119-70-6, 804-63-7 [anhydrous], 130-95-0	C20H26N2O6S	422.50	antimalarial, skeletal muscle relaxant	p10 3-3	-15.40	5.39	4	-0.12	0.06	4	-8.7

608	RHODINYL ACETATE	0	C12H22O2	198.31		P2 9-7	-15.58	8.00	4	0.17	0.15	4	10.9
609	AMYGDALIN	29883-15-6	C20H27NO11	457.44	antiinflammatory, experimental antineoplastic	p8 6-7	-15.61	20.76	4	0.07	0.04	4	4.8
610	UMBELLIFERONE	93-35-6	C9H6O3	162.15	antifungal, phytoalexin	p2 2-5	-15.67	2.30	4	-0.92	0.57	4	-88.9
611	IRIGENIN, DIBENZYL ETHER	0	C32H28O8	540.58		p9 8-2	-15.69	8.14	4	0.00	0.07	4	0.0
612	15-NORCARYOPHYLLEN-3-ONE	0	C14H22O	206.33		p6 8-4	-15.72	5.09	4	-0.05	0.11	4	-3.6
613	S-ISOCORYDINE (+)	475-67-2	C20H23NO4	341.41	sedative, cholinergic	p1 9-4	-15.72	13.54	4	-0.56	0.99	4	-47.4
614	DICTAMNINE	484-29-7	C12H9NO2	199.21		p3 3-4	-15.89	10.75	4	0.06	0.03	4	3.8
615	METHYL 7-DESOXYPURPUROGALLIN-7-CARBOXYLATE TRIMETHYL ETHER	0	C16H16O6	304.30		p9 3-3	-15.95	3.03	4	0.00	0.08	4	0.1
616	PERILLIC ACID (-)	7694-45-3	C10H14O2	166.22	inhibits posttranslational cys isoprenylation, blocks G-protein	p5 10-6	-16.19	10.71	4	0.14	0.16	4	8.9
617	MELIBIOSE	585-99-9	C12H22O11	342.30		p6 7-2	-16.19	7.01	4	-0.06	0.07	4	-4.0
618	N-ACETYLMURAMIC ACID	10597-89-4	C11H19NO8	293.28		p6 2-3	-16.52	6.87	4	-0.11	0.08	4	-8.2
619	DIGITONIN	11024-24-1	C56H92O29	1229.34		p8 4-2	-16.58	11.21	4	-0.12	0.03	4	-8.8
620	MELEZITOSE	0	C18H32O16	504.45		p9 3-1	-16.81	5.47	4	-0.18	0.06	4	-13.0
621	CHRYSANTHEMYL ALCOHOL	5617-92-5	C10H18O	154.25		p9 4-1	-16.89	6.13	4	-0.13	0.02	4	-9.5
622	7,2'-DIHYDROXYFLAVONE	77298-66-9	C15H10O4	254.24	antihaemorrhagic	p8 5-8	-16.96	7.55	4	0.19	0.09	4	12.6
623	gamma-AMINOBUTYRIC ACID	56-12-2	C4H9NO2	103.12	antihypertensive	p6 5-5	-17.05	6.18	4	0.04	0.03	4	2.6
624	NONIC ACID	0	C9H16O4	188.23		p5 8-3	-17.10	4.83	4	0.06	0.03	4	3.9
625	7-METHOXYCHROMONE	0	C10H8O3	176.17		p3 10-1	-17.15	5.76	4	-0.34	0.04	4	-26.1
626	TETRACYCLINE HYDROCHLORIDE	64-75-5, 60-54-8	C22H25ClN2O8	480.91	antibacterial, antiamebic, antirickettsial	p10 8-3	-17.26	6.52	4	-0.31	0.02	4	-24.3
627	LACTOBIONIC ACID	96-82-2	C12H22O12	358.30	food additive	p3 7-2	-17.48	3.56	4	-0.35	0.09	4	-27.5
628	GOSSYPETIN	489-35-0	C15H10O8	318.24		p5 5-2	-17.52	7.12	4	-0.04	0.08	4	-2.6
629	MYOSMINE	532-12-7	C9H10N2	146.19	mitogen	p6 5-3	-17.55	3.79	4	0.07	0.03	4	4.5
630	JUGLONE	481-39-0	C10H6O3	174.16	antineoplastic, antifungal	p7 8-6	-17.67	5.66	4	0.17	0.04	4	11.2
631	STIGMASTEROL	0	C29H48O	412.71		p1 4-6	-17.97	27.51	4	0.96	0.10	4	48.6
632	EMODIN	518-82-1	C15H10O5	270.24	antibacterial, antineoplastic, cathartic, tyrosine kinase inhibitor	p3 6-3	-17.99	7.24	4	-0.04	0.07	4	-3.0
633	1-HYDROXY-3,6,7-TRIMETHOXY-2,8-DIPRENYLXANTHONE	15404-76-9	C26H30O6	438.53		p3 4-6	-18.05	4.77	4	0.12	0.15	4	7.7
634	THEOBROMINE	83-67-0	C7H8N4O2	180.17	diuretic, bronchodilator, cardiotonic	P2 8-3	-18.14	6.74	4	0.03	0.09	4	2.2
635	GALANGIN TRIMETHYL ETHER	26964-29-4	C18H16O5	312.33		p3 9-2	-18.22	2.52	4	0.13	0.11	4	8.8
636	ISOTECTORIGENIN, 7-METHYL ETHER	0	C18H16O6	328.32		p4 10-5	-18.66	5.87	4	0.04	0.05	4	3.0
637	ASTRAGALOSIDE IV	84687-43-4	C41H68O14	784.99		p1 5-8	-18.98	33.36	4	-0.19	0.07	4	-14.1
638	2',4'-DIHYDROXYCHALCONE 4'-GLUCOSIDE	0	C21H22O8	402.40	anthelmintic & antiulcerocenic	p1 4-8	-19.50	24.66	4	0.03	0.19	4	1.9
639	AVOCADYNOFURAN	24708-33-6	C17H26O	246.40		p5 7-2	-19.78	21.92	4	0.02	0.10	4	1.4
640	PEONIFLORIN	23180-57-6	C23H30O11	482.49	antiinflammatory, antispasmodic, antihypertensive, antidiuretic	p8 3-4	-19.87	6.80	4	-0.11	0.10	4	-7.6
641	KAINIC ACID	487-79-6	C10H15NO4	213.24	glutamate receptor agonist, anthelmintic	p7 7-2	-19.89	9.21	4	-0.04	0.08	4	-2.6
642	HELENINE	546-43-0	C15H20O2	232.33	anthelmintic, antibacterial, antineoplastic	p7 5-5	-19.97	8.59	4	0.10	0.10	4	6.4
643	LECANORIC ACID	480-56-8	C16H14O7	318.29		p4 5-5	-19.97	8.59	4	-0.02	0.10	4	-1.2
644	3beta-HYDROXY-23,24-BISNORCHOL-5-ENIC ACID	0	C22H34O3	346.51		p3 5-1	-20.26	8.34	4	-0.71	0.01	4	-63.2
645	MADECASSIC ACID	18449-41-7	C30H48O6	504.71	wound healing	p1 9-8	-20.30	17.06	4	-0.16	0.13	4	-11.7
646	THREONINE (L)	72-19-5	C4H9NO3	119.12	amino acid, nutrient	p7 10-1	-20.50	11.38	4	-0.26	0.10	4	-19.5
647	HISTAMINE DIHYDROCHLORIDE	51-45-6 [histamine]	C5H11Cl2N3	184.07	H1&2 agonist, edema induction, gastric secretion stimulant	p10 3-2	-20.59	14.84	4	0.02	0.08	4	1.3
648	NEROL	106-25-2	C10H18O	154.25	weak estrogen receptor blocker	p5 9-6	-20.59	7.59	4	0.43	0.09	4	25.8
649	PROTOPORPHYRIN IX	553-12-8	C34H34N4O4	562.67	hepatoprotectant	p8 2-6	-20.60	28.20	4	0.00	0.20	4	-0.1
650	PTAEROXYLIN	14729-11-4	C15H14O4	258.28		p4 7-3	-20.69	5.56	4	-0.13	0.08	4	-9.7
651	XANTHOXYLIN	90-24-4	C10H12O4	196.20		p3 4-5	-20.73	2.33	4	0.08	0.04	4	5.4
652	VINCAMINE	1617-90-9	C21H26N2O3	354.45	vasodilator	p6 5-4	-20.79	8.57	4	0.12	0.06	4	8.2
653	NORSTICTIC ACID	571-67-5	C18H12O9	372.29	antibacterial	p6 2-1	-20.79	12.81	4	-0.18	0.09	4	-13.4
654	SINAPIC ACID METHYL ETHER	90-50-6	C12H14O5	238.24		p1 5-5	-20.82	14.35	4	0.66	0.12	3	36.6
655	PECTOLINARIN	28978-02-1	C29H34O15	622.59		p4 9-5	-20.96	9.20	4	1.99	1.75	4	74.8

656	YOHIMBIC ACID HYDRATE	522-87-2	C20H26N2O4	358.44		p6 2-5	-21.23	3.47	4	-0.27	0.06	4	-20.7
657	2',4-DIHYDROXYCHALCONE	13323-66-5	C15H12O3	240.26		p5 10-3	-21.40	13.06	4	0.13	0.13	4	8.3
658	AVOCADENOFURAN	25346-24-1	C17H28O	248.41		p6 6-3	-21.47	25.05	4	-0.41	0.09	4	-32.5
659	GOSSYPOL-ACETIC ACID COMPLEX	0	C32H34O10	578.62	male contraceptive	p6 10-1	-21.55	10.62	4	-0.10	0.06	4	-7.1
660	GANGALEOIDIN	55365-63-4	C18H14Cl2O7	413.21		p8 4-5	-21.59	19.44	4	-0.05	0.18	4	-3.3
661	DEHYDRODIHYDROROTENONE	6659-45-6	C23H22O6	394.43		P2 8-7	-21.64	13.80	4	0.06	0.12	4	4.4
662	BACITRACIN	1405-87-4	C66H103N17O16S	1422.73	antibacterial	p7 8-2	-21.81	8.19	4	0.02	0.06	4	1.4
663	OSAJIN 4'-METHYL ETHER	27762-88-5	C26H26O5	418.49		p8 5-7	-21.87	15.35	4	-0.04	0.06	4	-2.7
664	MIMOSINE	0	C8H10N2O4	198.18	depilatory agent	p8 4-8	-22.01	14.13	4	-0.01	0.11	4	-0.4
665	GOSSYPIN	652-78-8	C21H20O13	480.39		p5 5-5	-22.05	9.68	4	-0.07	0.08	4	-4.8
666	BERGAPTEN	484-20-8	C12H8O4	216.20	antipsoriatic, antiinflammatory	p6 3-5	-22.30	11.50	4	0.06	0.05	4	4.3
667	LANOSTEROL	79-63-0	C30H50O	426.73		p9 6-4	-22.47	9.27	4	-0.24	0.23	4	-18.1
668	CEAROIN	52811-37-7	C14H12O4	244.25		p8 8-3	-22.53	5.49	4	-0.27	0.20	4	-21.0
669	LYSINE (L) HYDROCHLORIDE	657-27-2	C6H15ClN2O2	182.65	amino acid, nutrient	p9 5-4	-22.55	8.13	4	-0.15	0.04	4	-11.0
670	TANNIC ACID	1401-55-4	C76H52O46	1701.23	nonspecific enzyme/receptor blocker	p6 5-8	-22.61	8.28	4	0.27	0.05	4	17.0
671	GUAIOL(-)	489-86-1	C15H26O	222.37		p6 8-2	-22.71	13.37	4	0.20	0.09	4	12.7
672	PROLINE (L)	147-85-3	C5H9NO2	115.13	amino acid, nutrient	p9 9-2	-22.72	4.93	4	0.03	0.06	4	1.9
673	CHOLESTERYL ACETATE	604-35-3	C29H48O2	428.70		p8 2-3	-22.86	10.92	4	-0.08	0.09	4	-5.8
674	3,4'-DIMETHOXYFLAVONE	0	C17H14O4	282.30		p4 10-7	-22.87	7.48	4	0.36	0.06	4	22.3
675	PUERARIN	3681-99-0	C21H20O9	416.39	beta-adrenergic blocker	p2 2-6	-22.97	9.51	4	-0.46	0.56	4	-37.4
676	ISOPIMPINELLIN	482-27-9	C13H10O5	246.22		p3 2-2	-23.17	11.47	4	-0.24	0.01	4	-18.1
677	HARMANE	486-84-0	C12H10N2	182.23	intercalating agent, sedative	p6 9-3	-23.18	8.01	4	0.07	0.03	4	4.5
678	5alpha-CHOLESTANOL	80-97-7	C27H48O	388.68		p3 5-4	-23.23	4.52	4	-0.03	0.05	4	-2.4
679	LINALOOL (+)	0	C10H18O	154.25		p6 10-4	-23.26	4.26	4	0.09	0.10	4	6.1
680	HYDRASTINE (1R, 9S)	118-08-1	C21H21NO6	383.40	antihypertensive, sedative, antibacterial	p10 10-4	-23.62	10.12	4	-0.18	0.04	4	-13.2
681	VIOLASTYRENE	19034-96-9	C17H18O3	270.33		p5 3-3	-23.64	6.82	4	-0.30	0.14	4	-23.3
682	4-METHYLDAPHNETIN	2107-77-9	C10H8O4	192.17		p8 1-7	-23.67	8.52	4	-0.22	0.03	4	-16.6
683	ESCULETIN	305-01-1	C9H6O4	178.15	antifungal	p1 2-4	-23.80	3.13	4	0.21	0.14	4	13.5
684	2',5'-DIHYDROXY-4-METHOXYCHALCONE	6342-92-3	C16H14O4	270.29		p5 2-3	-23.93	5.92	4	-0.46	0.08	4	-37.7
685	METHYL TRIMETHOXYCINNAMATE	7560-49-8, 20329-96-8	C13H16O5	252.27		p6 5-1	-24.35	9.66	4	0.04	0.03	4	2.8
686	KOBUSONE	24173-71-5	C14H22O2	222.33		p1 2-8	-24.42	17.62	4	0.01	0.10	4	0.5
687	DOCONEXENT	6217-54-5	C22H32O2	328.50	PAF inhibitor	p6 7-5	-24.46	18.07	4	-0.06	0.05	4	-4.0
688	DEHYDROCHOLIC ACID	81-23-2	C24H34O5	402.54	choleretic	p10 9-5	-24.47	17.89	4	-0.06	0.03	4	-4.2
689	CHLOROGENIC ACID	327-97-9	C16H18O9	354.32	antioxidant, free radical scavenger	p9 2-3	-24.50	8.41	4	-0.13	0.02	4	-9.1
690	TRIPTOPHENOLIDE	74285-86-2	C20H24O3	312.41		p9 6-5	-24.54	10.22	4	-0.29	0.16	4	-22.1
691	2-METHOXY-5 (6)EPOXY-TETRAHYDROCARYOPHYLLENE	0	C16H28O2	252.40		p3 9-1	-24.74	6.54	4	-0.15	0.05	4	-11.1
692	CARMINIC ACID	1260-17-9	C22H20O14	508.40		p5 8-2	-24.88	10.47	4	-0.10	0.11	4	-7.5
693	7-OXOCHOLESTERYL ACETATE	0	C29H46O3	442.69		p6 2-4	-24.94	4.48	4	-0.20	0.04	4	-15.2
694	PHLORACETOPHENONE	480-66-0	C8H8O4	168.15		P2 7-3	-25.30	8.69	4	0.44	0.24	3	26.5
695	ARTHONIOIC ACID	25556-24-5	C29H36O9	528.60		p3 4-2	-25.37	14.37	4	0.02	0.09	4	1.1
696	SOLANESOL	13190-97-1	C45H74O	631.09		p5 10-4	-25.87	1.79	4	0.16	0.12	4	10.5
697	GENETICIN	49863-47-0, 108321-42-	C20H40N4O10	496.56	antibacterial	p5 9-4	-26.05	16.30	4	0.07	0.07	4	4.9
698	RIBOFLAVIN	83-88-5	C17H20N4O6	376.37	Vitamin B2; Vitamin cofactor; LD5(rat) 56 mg/kg ip	p6 9-7	-26.60	14.48	4	0.02	0.03	4	1.1
699	HAEMATOXYLIN PENTAACETATE	0	C26H24O12	528.47		p5 7-4	-26.73	11.62	4	0.19	0.12	4	12.3
700	CHOLESTEROL	57-88-5	C27H46O	386.67	emulsifying agent	p7 4-2	-26.79	5.06	4	-0.05	0.03	4	-3.8
701	IRIGINOL HEXAACETATE	0	C27H22O14	570.47		p4 4-2	-26.79	5.06	4	-0.17	0.03	4	-12.3
702	ACETOSYRINGONE	2478-38-8	C10H12O4	196.20	insect attractant, plant hormone	p7 4-4	-26.85	11.01	4	-0.06	0.07	4	-4.0
703	ANGOLENSIN (R)	4842-48-2	C16H16O4	272.30		p4 4-4	-26.85	11.01	4	-0.17	0.07	4	-12.5
704	DUARTIN (-)	52305-04-1	C18H20O6	332.36		p5 6-3	-26.87	16.43	4	-0.20	0.09	4	-15.3
705	DIMETHYLCAFFEIC ACID	14737-89-4	C11H12O4	208.22		p2 2-4	-27.15	4.30	4	-0.49	0.57	4	-40.0
706	EPINEPHRINE BITARTRATE	51-42-3	C13H19NO9	333.30	adrenergic agonist, bronchodilator, antiglaucoma agent	p8 3-1	-27.15	4.27	4	0.04	0.08	4	2.6
707	HAEMATOXYLIN	517-28-2	C16H14O6	302.29		p4 9-4	-27.24	2.78	4	0.00	0.03	4	0.1

708	TYRAMINE	51-67-2	C8H11NO	137.18	adrenergic agonist	p8 3-3	-27.42	13.79	4	-0.27	0.15	4	-20.9
709	4-METHYLIMIDAZOLE	822-36-6	C4H6N2	82.11		p6 3-2	-27.80	13.76	4	0.18	0.07	4	11.5
710	5alpha-CHOLESTAN-3beta-OL-6-ONE	0	C27H46O2	402.67		p2 1-6	-27.83	14.93	4	0.53	0.04	4	30.6
711	CADIN-4-EN-10-OL	0	C15H26O	222.37		p9 1-3	-28.21	9.30	4	-0.42	0.11	4	-34.1
712	SOLASODINE	126-17-0	C27H43NO2	413.65	antineoplastic, antiinflammatory	p3 10-4	-28.34	3.86	4	0.07	0.08	4	4.6
713	4-METHOXYDALBERGIONE	4646-86-0	C16H14O3	254.29		p1 10-8	-28.49	14.51	4	-0.03	0.11	4	-2.3
714	APHYLLIC ACID	642-67-1	C15H26N2O2	266.39		p1 10-7	-28.53	19.92	4	0.25	0.03	4	16.1
715	CARYLOPHYLLENE OXIDE	1139-30-6	C14H22O	206.33		p1 8-7	-28.53	19.92	4	-0.23	0.10	4	-17.5
716	BOLDINE	476-70-0	C19H21NO4	327.38		p9 1-2	-28.61	6.78	4	-0.34	0.12	4	-26.4
717	ERGOSTEROL	57-87-4	C28H44O	396.66		p4 10-2	-29.64	1.95	4	-0.03	0.07	4	-1.9
718	TRIGONELLINE	535-83-1	C7H7NO2	137.14	antihyperglycemic	P2 8-2	-29.89	12.83	4	0.03	0.06	4	2.2
719	PILOCARPINE NITRATE	148-72-1, 92-13-7	C11H17N3O5	271.28	antiglaucoma agent, miotic	p10 10-2	-30.24	2.97	4	-0.10	0.03	4	-7.3
720	HUMULENE (alpha)	6753-98-6	C15H24	204.36		p6 10-2	-30.91	4.06	4	0.00	0.03	4	0.1
721	GENTAMICIN SULFATE	1405-41-0, 1403-66-3	C21H45N5O11S	575.68	antibacterial	p10 2-2	-30.99	4.60	4	-0.07	0.07	4	-4.7
722	RHETSININE	526-43-2	C19H17N3O2	319.37		p2 6-1	-31.21	10.59	4	0.51	0.23	4	29.6
723	13-METHYL-4,4-BISNOR-8,11,13-PODOCARPATRIEN-3-ONE	0	C16H20O	228.34		p8 1-6	-31.37	15.11	4	-0.03	0.10	4	-2.0
724	HARMALINE	304-21-2	C13H14N2O	214.27	CNS stimulant, antiparkinsonian agent	p6 5-2	-31.87	10.11	4	0.01	0.03	4	0.9
725	DIHYDROXY (3alpha,12alpha)PREGNAN-20-ONE	0	C21H34O3	334.50		p8 9-5	-32.59	19.91	4	-0.37	0.05	4	-29.0
726	GUAIACOL	90-05-1	C7H8O2	124.14	expectorant	p6 10-3	-32.94	10.42	4	0.02	0.04	4	1.6
727	ISOKOBUSONE	24173-72-6	C14H22O2	222.33		p3 10-2	-33.53	3.68	4	-0.25	0.04	4	-18.7
728	SORBITOL	50-70-4	C6H14O6	182.17	sweetening agent and humectant	p6 3-4	-33.80	11.82	4	-0.18	0.06	4	-13.2
729	4,4'-DIMETHOXYDALBERGIONE	0	C17H16O4	284.31		p5 6-4	-34.16	35.02	4	-0.01	0.08	4	-0.7
730	POMIFERIN TRIMETHYL ETHER	0	C28H30O6	462.55	derivative	p9 9-3	-34.33	10.46	4	0.17	0.06	4	11.4
731	METHYL ORSELLINATE	3187-58-4	C9H10O4	182.18		p2 3-4	-34.82	13.79	4	0.31	0.07	4	19.6
732	beta-CAROTENE	7235-40-7	C40H56	536.89	antioxidant; provitamin A	p7 10-2	-34.82	5.87	4	0.04	0.07	4	2.8
733	BISPHENOL A	80-05-7	C15H16O2	228.29	endocrine disruptor, plastic monomer	p7 3-2	-35.16	16.89	4	-0.20	0.03	4	-15.0
734	DEACETOXY(7)-7-OXOKHIVORINIC ACID	0	C27H36O10	520.58		p4 3-2	-35.16	16.89	4	-0.32	0.03	4	-24.4
735	NEROLIDOL	7212-44-4	C15H26O	222.37		p5 3-7	-35.53	12.65	4	-0.17	0.16	4	-12.9
736	ACETYL HYMETOCHROME	2747-05-9	C12H10O4	218.21		p6 2-2	-35.81	14.76	4	-0.06	0.10	4	-4.5
737	RIBOSTAMYCIN SULFATE	25546-65-0	C17H36N4O14S	552.56	antibacterial	p9 5-2	-35.94	13.39	4	-0.03	0.23	4	-2.0
738	EPIANDROSTERONE	0	C19H30O2	290.45		p3 4-1	-36.02	4.97	4	-0.05	0.02	4	-3.8
739	CHOLESTAN-3-ONE	566-88-1	C27H46O	386.67		p5 2-6	-36.69	3.48	4	-0.48	0.06	4	-39.9
740	PURPURIN	81-54-9	C14H8O5	256.22	xanthin oxidase inhibitor, irritant	p3 7-3	-37.23	5.62	4	-0.19	0.02	4	-14.3
741	3,4-DIDESMETHYL-5-DESHYDROXY-3'-ETHOXYSCLEROIN	0	C15H14O5	274.28		p9 5-1	-37.23	10.14	4	-0.39	0.13	4	-31.4
742	RESVERATROL 4'-METHYL ETHER	33626-08-3	C15H14O3	242.28		p7 2-2	-37.33	11.06	4	-0.37	0.07	4	-29.0
743	FRAXIDIN METHYL ETHER	0	C12H12O5	236.23		p4 2-2	-37.33	11.06	4	-0.48	0.07	4	-39.5
744	CHOLEST-5-EN-3-ONE	601-54-7	C27H44O	384.65		p9 4-2	-37.48	6.58	4	0.04	0.04	4	2.7
745	APIGENIN DIMETHYL ETHER	5728-44-9	C17H14O5	298.30		p1 2-3	-37.84	16.95	4	0.17	0.16	4	11.3
746	beta-AMYRIN	559-70-6	C30H50O	426.73		p9 10-2	-38.19	9.19	4	-0.12	0.12	4	-8.3
747	GUAIAZULENE	489-84-9	C15H18	198.31	antioxidant, inhibits lipid peroxidation inhibitor, antiinflammatory, hepatoprotectant; LD5(rat) 155 mg/kg po	p8 6-3	-38.21	55.45	4	-0.34	0.16	4	-27.0
748	DEHYDROROTENONE	30990-44-4	C23H20O6	392.41		p5 5-3	-38.48	8.13	4	-0.07	0.08	4	-4.9
749	QUEBRACHITOL	642-38-6	C7H14O6	194.19		p9 3-2	-39.02	2.63	4	-0.16	0.06	4	-11.4
750	3-HYDROXYCOUMARIN	939-19-5	C9H6O3	162.15		p3 10-3	-39.59	7.07	4	-0.21	0.03	4	-15.7
751	DEOXYCHOLIC ACID	88-44-3	C24H40O4	392.58		p1 9-7	-39.82	12.56	4	0.55	0.28	4	31.7
752	FISSINOLIDE	1915-69-1	C29H36O8	512.61		p1 7-7	-39.82	12.56	4	-0.81	0.06	4	-75.3
753	3-ACETYLGEDUNOL	0	C30H40O8	528.65		p1 5-7	-40.49	7.59	4	1.19	0.12	3	56.3
754	21-ACETOXYPREGNENOLONE	566-78-9	C23H34O4	374.53	precursor in corticoid biosynthesis, derivative	p5 8-5	-41.17	5.68	4	0.10	0.10	4	6.5
755	URSODIOL	128-13-2	C24H40O4	392.58	anticholelithogenic; LD5(rat) 89 mg/kg ip	p10 10-3	-41.66	8.38	4	-0.15	0.05	4	-10.9
756	HARPAGOSIDE	19210-12-9	C23H28O11	480.47		p5 9-5	-42.12	2.29	4	0.36	0.09	4	21.8
757	ORNITHINE HYDROCHLORIDE	70-26-8	C5H13ClN2O2	168.62	hepatoprotectant, anticholesteremic	p6 10-5	-42.36	8.44	4	-0.07	0.12	4	-5.2
758	ALIZARIN	72-48-0	C14H8O4	240.22	antimutagen	p9 5-3	-42.36	4.93	4	-0.13	0.07	4	-9.1

759	KINETIN	525-79-1	C10H9N5O	215.22	auxin, plant growth regulator, plant cell division promotor	p1 6-4	-42.74	18.96	4	0.54	0.02	4	31.0
760	CITROPTEN	487-06-9	C11H10O4	206.20	photosensitizing agent	p2 6-2	-43.58	6.75	4	0.38	0.07	4	23.1
761	2,3,4-TRIHYDROXY-4'-ETHOXYBENZOPHENONE	0	C15H14O5	274.28		p9 10-8	-43.73	7.79	4	0.22	0.05	4	14.4
762	GRAMINE	87-52-5	C11H14N2	174.25		p8 7-4	-43.87	19.34	4	-0.42	0.13	4	-33.5
763	APIGENIN	520-36-5	C15H10O5	270.24	antispasmodic, antineoplastic, topoisomerase I inhibitor	p9 10-3	-44.57	6.62	4	0.06	0.05	4	3.9
764	METHYL ROBUSTONE	0	C22H18O6	378.39		P2 9-5	-44.64	13.43	4	-0.08	0.15	4	-5.4
765	CITRULLINE	627-77-0	C6H13N3O3	175.19		p1 5-4	-44.68	15.25	4	0.62	0.10	2	34.9
766	ATRANORIN	479-20-9	C19H18O8	374.35		p1 4-7	-45.91	11.98	4	1.02	0.16	4	50.7
767	LAWSONE	83-72-7	C10H6O3	174.16		p6 1-5	-46.08	13.52	4	-0.16	0.11	4	-12.0
768	PYRROMYCIN	668-17-7	C30H35NO11	585.61	antibacterial	p8 3-5	-46.08	5.01	4	0.35	0.15	4	21.5
769	DEFEROXAMINE MESYLATE	138-14-7, 70-51-9	C26H52N6O11S	656.80	chelating agent (Fe & Al)	p10 3-1	-48.55	15.61	4	0.31	0.07	4	19.6
770	LATHOSTEROL	0	C27H46O	386.67		p2 3-8	-50.10	20.42	4	0.35	0.08	4	21.4
771	HYDROXYPROGESTERONE	3168-01-2	C21H30O3	330.47	progestin	p6 9-4	-50.17	0.99	4	0.37	0.06	4	22.7
772	APIOLE	523-80-8	C12H14O4	222.24	antipyretic, diuretic, insecticide	p7 5-4	-50.23	2.73	4	0.06	0.04	4	3.8
773	3alpha-HYDROXY-4,4-BISNOR-8,11,13-PODOCARPATRIENE	0	C15H20O	216.33		p4 5-4	-50.23	2.73	4	-0.06	0.04	4	-4.1
774	GARCINOLIC ACID	0	C38H46O9	646.78		p2 3-2	-51.29	18.22	4	0.36	0.16	4	22.0
775	NOSCAPINE HYDROCHLORIDE	912-60-7, 128-62-1	C22H24ClNO7	449.89	antitussive	p10 2-4	-51.62	13.18	4	0.30	0.05	4	18.9
776	EPICATECHIN PENTAACETATE	0	C25H24O11	500.46		p6 3-1	-52.46	17.01	4	-0.02	0.01	4	-1.3
777	ALLOPREGNANOLONE	0	C21H34O2	318.50		p4 9-1	-52.47	4.62	4	0.02	0.08	4	1.3
778	EQUILIN	474-86-2	C18H20O2	268.36	estrogen	p10 6-1	-53.61	45.92	4	-0.60	0.10	4	-51.5
779	CAPSAICIN	404-86-4	C18H27NO3	305.42	analgesic (topical), depletes Substance P, neurotoxic	p10 1-4	-54.23	9.61	4	0.00	0.17	4	0.1
780	THEANINE POTASSIUM	3081-61-6	C7H13KN2O3	212.30		p3 5-3	-54.47	14.26	4	-0.45	0.07	4	-36.3
781	HARMOL HYDROCHLORIDE	40580-83-4	C12H11ClN2O	234.69	MAO inhibitor	p1 2-2	-55.14	10.17	4	-0.14	0.05	4	-10.1
782	LITHOCHOLIC ACID	434-13-9	C24H40O3	376.58	LD5(mouse) 39 mg/kg po	p1 2-5	-55.75	9.37	4	0.65	0.06	4	36.1
783	JUAREZIC ACID	1552-94-9	C11H10O2	174.20		p3 5-2	-55.89	17.98	4	-0.57	0.05	4	-48.4
784	PHYSCION	521-61-9	C16H12O5	284.27	antibacterial, cathartic	p9 9-5	-57.45	5.78	4	0.20	0.03	4	12.9
785	ABIETIC ACID	514-10-3	C20H30O2	302.46		p9 2-2	-58.27	13.29	4	-0.39	0.11	4	-30.7
786	GLYCOCHOLIC ACID	475-31-0	C26H43NO6	465.64		p8 6-4	-58.36	28.23	4	-0.31	0.30	4	-23.7
787	POMIFERIN DIMETHYL ETHER	0	C27H28O6	448.52		p9 4-3	-58.61	11.00	4	0.09	0.06	4	6.1
788	CORYNANTHINE	123333-62-0	C21H26N2O3	354.45		p8 7-7	-58.95	29.94	4	-0.61	0.06	4	-53.1
789	MELATONIN	73-31-4	C13H16N2O2	232.28	sleep induction, modifies circadian rhythm	p2 2-7	-62.19	3.45	4	-1.56	0.66	4	-195.5
790	TRIACETYLRESVERATROL	42206-94-0	C20H18O6	354.36		p3 4-3	-64.78	13.82	4	0.19	0.12	4	12.4
791	3-METHOXYCATECHOL	934-00-9	C7H8O3	140.14		p3 8-2	-66.69	9.30	4	0.05	0.04	4	3.2
792	CHRYSOPHANOL	481-74-3	C15H10O4	254.24		P2 9-4	-71.62	13.37	4	0.17	0.16	4	11.4
793	UBIDECARENEONE	303-98-0	C59H90O4	863.37	cardiovascular agent	p1 10-5	-74.78	16.33	4	0.35	0.15	4	21.3
794	7-DEACETYLKHIVORIN	0	C30H40O9	544.65		p1 8-5	-74.78	16.33	4	-0.55	0.06	4	-46.2
795	VULPINIC ACID	521-52-8	C19H14O5	322.32	antiinflammatory, antibacterial, plant growth inhibitor	p7 10-4	-77.92	7.79	4	0.64	0.09	4	36.0
796	FUSIDIC ACID	6990-06-3	C31H48O6	516.72	antibacterial	p10 1-2	-82.83	11.31	4	-0.30	0.14	4	-23.2
797	SINOMENINE	115-53-7	C19H23NO4	329.40	weak abortifacient, immunosuppressant, analgesic, antiinflammatory; LD5 (po) 58 mg/kg; (ip) 285 mg/kg(mouse)	p2 2-8	-83.45	48.85	4	-0.78	0.59	4	-71.8
798	QUERCETIN 5,7,3',4'-TETRAMETHYL ETHER	1244-78-6	C19H18O7	358.35		p8 7-3	-84.09	21.55	4	-0.81	0.33	4	-75.6
799	TANSHINONE IIA SULFONATE SODIUM	0	C19H17NaO6S	396.40	free radical scavenger	p7 3-3	-96.29	8.40	4	0.01	0.04	4	0.9
800	HETEROPEUCENIN, METHYL ETHER	26213-95-6	C16H18O4	274.32		p4 3-3	-96.29	8.40	4	-0.10	0.04	4	-7.2
