# Supplementary material for: Discovery of Novel Anti-cryptosporidial Activities From Natural Products by in vitro High-Throughput Phenotypic Screening
Source: Front Microbiol. 2019 Aug 29;10:1999. doi: 10.3389/fmicb.2019.01999 (PMC6736568; doi:10.3389/fmicb.2019.01999)
Supplement: Supplementary file 2 [file Table_2.pdf]

Table S2. Secondary screening: Anti-cryptosporidial activity of 88 compounds at 3.3 uM.

#	Compound name	CAS #	Formula	Mol Wt	Bioactivity and note	Plate #	% Inhibition (3.3 uM)	SEM	N
1	MONENSIN SODIUM (monensin A is shown)	22373-78-0, 17090-79-8 (monensin)	C37H63NaO10	690.89846	antibacterial	P10 6-6	99	0.22	4
2	DACTINOMYCIN	50-76-0	C62H86N12O16	1255.44752	antineoplastic, intercalating agent	P6 10-6	97	0.59	4
3	EMETINE DIHYDROCHLORIDE	316-42-7, 483-18-1 [emetine]	C29H42Cl2N2O4	553.57509	inhibits RNA, DNA and protein synthesis	P2 1-4	95	0.75	4
4	PACLITAXEL	33069-62-4	C47H51NO14	853.92882	antineoplastic	P10 2-7	93	0.56	4
5	CHRYSANTHEMIC ACID, ETHYL ESTER		C12H20O2	196.292	insecticide	P1 6-1	91	1.72	4
6	VALINOMYCIN	2001-95-8	C54H90N6O18	1111.3488	antibiotic; LD50 (rat, po) 4 mg/kg	P7 4-1	87	2.06	4
7	MITOMYCIN	50-07-7	C15H18N4O5	334.33451	antineoplastic	P7 9-2	78	2.14	4
8	CYCLOSPORINE	59865-13-3	C62H111N11O12	1202.64247	immunosuppressant	p10 2-6	77	0.95	4
9	DEACETOXY-7-OXOGEDUNIN		C26H30O6	438.5254		p3 8-8	77	3.50	4
10	ROTENONE	83-79-4	C23H22O6	394.42819	acaricide, ectoparasiticide, antineoplastic, mitochondrial poison	P8 5-5	76	3.38	4
11	DIHYDROTANSHINONE I		C18H14O3	278.31048		p5 7-6	75	8.45	4
12	3-DEOXO-3beta-HYDROXYMEXICANOLIDE 16-ENOL ETHER		C28H36O7	484.59492		P4 6-1	71	3.02	4
13	DEOXYSAPPANONE B 7,3'-DIMETHYL ETHER ACETATE		C20H20O6	356.3788		p5 4-4	71	3.56	4
14	DAUNORUBICIN	20830-81-3	C27H29NO10	527.53288	antineoplastic	p10 2-1	71	4.88	4
15	DIGOXIGENIN	1672-46-4	C23H34O5	390.52443		P3 6-5	69	1.40	4
16	VINBLASTINE SULFATE	143-67-9, 865-21-4 [vinblastine]	C46H60N4O13S	909.0741	antineoplastic, spindle poison	p6 2-7	67	6.71	4
17	BAICALEIN	491-67-8	C15H10O5	270.24395	antiviral (HIV)	p3 3-3	66	2.22	4
18	PODOPHYLLIN ACETATE	1180-34-3	C24H24O9	456.45348		p5 3-6	64	5.29	4
19	8beta-HYDROXYCARAPIN, 3,8-HEMIACETAL		C27H32O8	484.55129		P3 5-7	64	3.16	4
20	TANSHINONE IIA	568-72-9	C19H18O3	294.35351	antineoplastic, bone resorption inhibitor, antiproliferative, apoptosis inducer	P8 4-4	63	2.87	4
21	DIMETHYLSULFONE	67-71-0	C2H6O2S	94.13292	antiinflammatory, antiproliferative, antiparasitic	p7 8-3	61	8.34	4
22	CAMPTOTHECIN	7689-03-4	C20H16N2O4	348.36152	antineoplastic	p5 5-7	59	3.04	4
23	2,3,4'-TRIHYDROXY-4-METHOXYBENZOPHENONE		C14H12O5	260.24874		p5 10-2	59	7.86	4
24	LOVASTATIN	75330-75-5	C24H36O5	404.55152	antihyperlipidemic, HMGCoA reductase inhibitor	p2 1-3	58	3.06	4
25	DIHYDROGAMBOGIC ACID		C38H46O8	630.78552		P1 4-3	57	5.70	4
26	PICROPODOPHYLLIN	477-47-4	C22H22O8	414.41584	Insulin growth factor 1 receptor inhibitor, antineoplastic	p9 7-4	56	3.66	4
27	DEACETYLGEDUNIN		C26H32O6	440.54134		P4 10-6	56	6.00	4
28	DEOXYSAPPANONE B 7,4'-DIMETHYL ETHER		C18H18O5	314.34116		P4 8-6	52	5.61	4
29	3,16-DIDEOXYMEXICANOLIDE-3beta-DIOL		C27H36O7	472.58377		p6 9-1	52	11.09	4
30	DIMETHYL GAMBOGINATE		C40H49ClO8	693.28473		P6 7-1	47	6.66	4
31	ISOOSAJIN	5745-54-0	C25H24O5	404.46703		P5 1-4	39	10.37	4
32	HESPERIDIN	520-26-3	C28H34O15	610.57418	capillary protectant	p7 7-8	36	11.29	4
33	SIROLIMUS	53123-88-9	C51H79NO13	914.19718	immunosuppressant, antineoplastic; rapamycin	p10 4-7	35	5.34	4
34	BUSSEIN	41060-14-4	C43H54O18	858.89903		P4 5-2	34	6.63	4
35	HAEMATOPORPHYRIN	14459-29-1	C34H38N4O6	598.70516	antidepressant, antineoplastic	P8 9-4	33	9.67	4

36	PRISTIMERIN	1258-84-0	C30H40O4	464.6509	antineoplastic, antiinflammatory	P3 8-5	30	8.13	4
37	DEMETHYLNOBILETIN	2174-59-6	C20H20O8	388.3776		P1 6-8	29	4.47	4
38	ACETYL ISOGAMBOGIC ACID		C40H46O9	670.80722		P3 7-5	27	6.35	4
39	PHENYLALANIINE (L) HYDROCHLORIDE	63-91-2(base)	C9H12ClNO2	201.65449	amino acid	p10 5-5	26	7.80	4
40	3alpha-ACETOXYDIHYDRODEOXYGEDUNIN		C30H40O7	512.6491		P4 4-1	25	6.41	4
41	TOMATINE	86273-92-9	C47H79NO21	994.14778	antifungal, antibacterial, antiinflammatory agent	P9 3-6	25	9.90	4
42	RESERPINE	50-55-5	C33H40N2O9	608.69475	antihypertensive	P10 4-3	24	2.75	4
43	beta-SITOSTEROL	83-46-5	C29H50O	414.72125		P8 8-5	20	8.93	4
44	STROPHANTHIDINIC ACID LACTONE ACETATE		C25H32O7	444.52959		P3 4-4	19	7.91	4
45	URIDINE TRIPHOSPHATE TRISODIUM	19817-92-6	C9H12N2Na3O15P3	550.09119	psychostimulant	P8 2-1	17	12.69	4
46	TRYPTOPHAN	73-22-3 ['L']	C11H12N2O2	204.23049	antidepressant, nutrient; LD50(rat) 1634 mg/kg ip	p1 7-6	17	2.94	4
47	ESTRAGOLE	140-67-0	C10H12O	148.20654	insect atttractant, skin irritant, carcinogen	P8 2-4	15	6.83	4
48	BENZYL ISOTHIOCYANATE	622-78-6	C8H7NS	149.21569	antineoplastic, antibacterial, antifungal	P9 1-8	14	9.03	4
49	PLUMBAGIN	481-42-5	C11H8O3	188.18461	antibacterial, antifungal, tuberculostatic; antifeedant (worm)	P5 10-5	13	4.05	4
50	TRYPTAMINE	61-54-1	C10H12N2	160.22054	psychotropic	P8 7-2	12	4.44	4
51	PIPLARTINE	20069-09-4	C17H19NO5	317.34468	anti-asthma, antibronchitis	p5 5-1	10	20.67	4
52	PATULIN	149-29-1	C7H6O4	154.12347	antibacterial	P9 10-4	10	8.80	4
53	GRISEOFULVIN	126-07-8	C17H17ClO6	352.77444	antifungal, inhibits mitosis in metaphase	p10 6-8	9	8.34	4
54	SAFROLE	94-59-7	C10H10O2	162.19	anesthetic (topical) and antiseptic, pediculicide	P9 3-8	8	16.16	4
55	3-HYDROXYTYRAMINE	62-31-7	C8H11NO2	153.18237	dopaminergic	P1 9-6	6	7.11	4
56	IVERMECTIN	70288-86-7	C48H74O14	875.11658	antiparasitic	p10 1-8	6	9.34	4
57	1,3-DIDEACETYL-7-DEACETOXY-7-OXOKHIVORIN		C26H34O7	458.55668		p3 7-4	3	16.46	4
58	TETRANDRINE	518-34-3	C38H42N2O6	622.76824	analgesic, antineoplastic, antihypertensive, lymphotoxin	P9 1-7	1	9.68	4
59	RUTILANTINONE	21288-61-9	C22H20O9	428.3993	coccidiostat	P9 2-6	-3	8.36	4
60	QUINIC ACID	77-95-2	C7H12O6	192.17009		p7 6-8	-6	12.67	4
61	STIGMASTA-4,22-DIEN-3-ONE	20817-72-5	C29H46O	410.68937		p7 9-8	-6	12.53	4
62	OCTOPAMINE HYDROCHLORIDE	104-14-3	C8H12ClNO2	189.64334	adrenergic agonist	P7 5-2	-12	7.63	4
63	TETRAHYDROGAMBOGIC ACID		C38H48O8	632.80146		P9 2-8	-12	9.60	4
64	ERYTHROMYCIN	114-07-8	C37H67NO13	733.94544	antibacterial	P6 2-8	-13	10.97	4
65	TACROLIMUS	109581-93-3, 104987-11-3 [anhydrous]	C44H69NO12	804.04003	immune suppressant, antifungal	p10 3-7	-14	21.93	4
66	ABAMECTIN (avermectin B1a shown)	71751-41-2	C48H72O14	873.10064	antiparisitic	P8 3-6	-19	7.04	4
67	GLUTATHIONE	70-18-8	C10H17N3O6S	307.32749	antioxidant	P9 5-8	-20	17.40	4
68	3beta-HYDROXYDEOXODIHYDRODEOXYGEDUNIN		C28H38O6	470.61146		p4 7-4	-21	22.23	4
69	RESVERATROL	501-36-0	C14H12O3	228.24994	antifungal, antibacterial	P4 1-2	-24	34.67	4
70	NOBILETIN	478-01-3	C21H22O8	402.40469	matrix metaloproteinase inhibitor; antineoplastic	P5 7-1	-25	17.26	4
71	QUERCETIN	117-39-5, 6151-25-3(hydrate)	C15H10O7	302.24275	capillary protectant, antioxidant. antineoplastic, anti-HIV	P7 7-4	-27	15.75	4
72	3-HYDROXYFLAVONE	577-85-5	C15H10O3	238.24515		p8 3-8	-28	8.03	4
73	SILIBININ	22888-70-6	C25H22O10	482.44809	hepatoprotective agent, antioxidant	P7 1-2	-30	37.73	4

74	HEXAMETHYLQUERCETAGETIN	1251-84-9	C21H22O8	402.40469		P3 1-7	-42	13.24	4
75	MUNDULONE	481-94-7	C26H26O6	434.49352		p4 10-4	-43	18.51	4
76	CAPREOMYCIN SULFATE	1405-37-4, 11003-38-6 [capreomycin]	C25H46N14O12S	766.79597	antibacterial, tuberculostatic	P7 2-8	-58	30.18	4
77	HARMINE	442-51-3	C13H12N2O	212.25339	antiparkinsonian, CNS stimulant	p9 4-8	-62	18.91	4
78	DIHYDROCELASTROL		C29H40O4	452.63975		P1 7-1	-66	10.44	4
79	CHRYSIN DIMETHYL ETHER	21392-57-4	C17H14O4	282.29873		P8 8-6	-69	15.08	4
80	ASARYLALDEHYDE	4460-86-0	C10H12O4	196.20474	fly attractant	P8 6-5	-74	14.55	4
81	DERRUSTONE	2204-59-3	C18H14O6	326.30868		p7 6-7	-77	35.57	4
82	5-HYDROXY-2',4',7,8-TETRAMETHOXYFLAVONE	123316-61-0	C19H18O7	358.35111		P6 5-7	-83	36.20	4
83	CYCLOVERATRYLENE		C27H30O6	450.53655		P8 1-5	-85	51.07	4
84	AVERMECTIN A1a		C49H74O14	887.12773	antiparasitic	P5 1-7	-85	35.38	4
85	CRYPTOTANSHINONE	35825-57-1	C19H20O3	296.36945	inhibits angiogenesis	P4 2-8	-85	9.62	4
86	OBTUSAQUINONE	21105-15-7	C16H14O3	254.28818		p4 6-5	-92	40.91	4
87	LAPACHOL	84-79-7	C15H14O3	242.27703	antineoplastic, antifungal	P8 8-8	-98	8.34	4
88	4-NONYLPHENOL	104-40-5	C15H24O	220.35793	weevil pheromone, shows estrogenic activity	p9 9-8	-181	41.78	4
